# Learning the structure of the world: The adaptive nature of state-space and action representations in multi-stage decision-making

**DOI:** 10.1371/journal.pcbi.1007334

**Published:** 2019-09-06

**Authors:** Amir Dezfouli, Bernard W. Balleine

**Affiliations:** 1 School of Psychology, UNSW, Sydney, Australia; 2 Data61, CSIRO, Sydney, Australia; Dartmouth College, UNITED STATES

## Abstract

State-space and action representations form the building blocks of decision-making processes in the brain; states map external cues to the current situation of the agent whereas actions provide the set of motor commands from which the agent can choose to achieve specific goals. Although these factors differ across environments, it is currently unknown whether or how accurately state and action representations are acquired by the agent because previous experiments have typically provided this information a priori through instruction or pre-training. Here we studied how state and action representations adapt to reflect the structure of the world when such a priori knowledge is not available. We used a sequential decision-making task in rats in which they were required to pass through multiple states before reaching the goal, and for which the number of states and how they map onto external cues were unknown a priori. We found that, early in training, animals selected actions as if the task was not sequential and outcomes were the immediate consequence of the most proximal action. During the course of training, however, rats recovered the true structure of the environment and made decisions based on the expanded state-space, reflecting the multiple stages of the task. Similarly, we found that the set of actions expanded with training, although the emergence of new action sequences was sensitive to the experimental parameters and specifics of the training procedure. We conclude that the profile of choices shows a gradual shift from simple representations to more complex structures compatible with the structure of the world.

## Author summary

Everyday decision-making tasks typically require taking multiple actions and passing through multiple states before reaching desired goals. Such states constitute the state-space of the task. Here we show that, contrary to current assumptions, the state-space is not static but rather expands during training as subjects discover new states that help them efficiently solve the task. Similarly, within the same task, we show that subjects initially only consider taking simple actions, but as training progresses the set of actions can expand to include useful action sequences that reach the goal directly by passing through multiple states. These results provide evidence that state-space and action representations are not static but are acquired and then adapted to reflect the structure of the world.

## Introduction

In sequential decision-making tasks, an agent makes a series of choices and passes through several states before earning rewards. Making choices requires knowing the structure of the environment such as the available actions, the number of states of the environment and how they map on to external cues, i.e., the *state-space* of the task. Within the lab this information is typically given to subjects in advance of the task either through instructions or pre-training, however, such a priori information is not usually made available to a decision-maker in natural environments, and the decision-making process must, therefore, involve (i) learning the correct state-space of the environment and (ii) acquiring new actions that are useful for earning reward in the task.

Learning the state-space of the task is crucial in allowing the agent to navigate within the environment, and provides building blocks for various forms of reinforcement-learning algorithms in the brain [1, 2]. This process involves considering different events and cues that occur after taking each action, and integrating them in order to recover how many states the task has and how they are related to external cues. Recent theoretical work provides a basis for this process in the context of classical conditioning and suggests that the underlying states used to represent the environment are dynamic; that animals are able to infer and learn new states of the environment based on their observations [3, 4]. However, at present, there is no direct evidence for such adaptive state-space representations in decision-making situations.

Actions are the other building block for reinforcement-learning algorithms, and refer, for example, to different motor commands that an agent can use to influence the state of the environment. Efficient decision-making relies on using actions at the appropriate scale; engaging decision-making at too fine-grained a level of motor movement will overwhelm this process with choice points. Indeed, evidence suggests that humans and other animals can create new actions in the form of action chunks or action sequences by concatenating simple actions together [5, 6, 7, 8, 2]. Such action sequences, known as ‘temporally extended actions’, can be thought of as new skills that expand the set of available actions and that are treated as single response units. By acquiring new action sequences, the selection process needs to be implemented only once at the initiation point of an action sequence instead of before each individual action and, in this way, adaptive representations of actions contribute to the scalability of the decision-making process.

In the current study, using a sequential decision-making task in rats, we sought to investigate whether state-space and action representations adapt to the structure of the world. We used a two-stage decision-making task similar to a two-stage task previously used in human subjects and rodents [e.g., 9, 10, 11, 12], and show, without any explicit instructions about the structure of the task (which obviously cannot be provided to rats), that early in training, the rats made decisions based on the assumption that the state-space is simple and the environment is composed of a single stage whereas, later in training, they learned the true state-space reflecting the multi-stage structure of the environment and made decisions accordingly. We also found that concurrently with the expansion of the state-space, the set of actions also expanded and action sequences were added to the set of actions that the rats executed. This latter effect, however, was sensitive to the choice of experimental parameters and did not emerge in all of the test conditions. We conclude that decision making is adaptive and depends on acquiring, refining and updating both state-space and action representations over time. Importantly, although this may not seem an entirely unexpected result from an associative learning perspective [e.g., 13], it required the development of an alternative hierarchical architecture to generate a description of the rats’ performance in RL terms.

## Results

**Fig 1:**
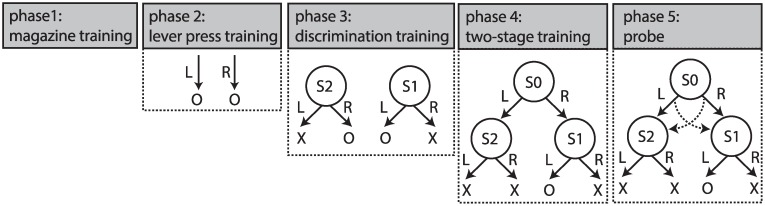
Different phases of the experiment. The experiment started with two magazine training sessions (phase 1), followed by several lever training sessions (phase 2), in which animals learned that pressing each lever (left and right levers corresponding to ‘L’ and ‘R’ in the figure) would delivered a reward (presented by ‘O’ in the figure). The next phase was discrimination training (phase 3), in which animals learned that when stimulus S1 was presented action ‘L’ should be taken to earn a reward, and when S2 was presented action ‘R’ should be taken to earn a reward. S1 and S2 were a constant and blinking house light, respectively. The next phase of the experiment was two-stage training, in which animals were trained on a two-stage decision-making task. This training phase comprised multiple training sessions and, in the middle or at the end of these training sessions, several ‘probe sessions’ were inserted.

**Fig 2:**
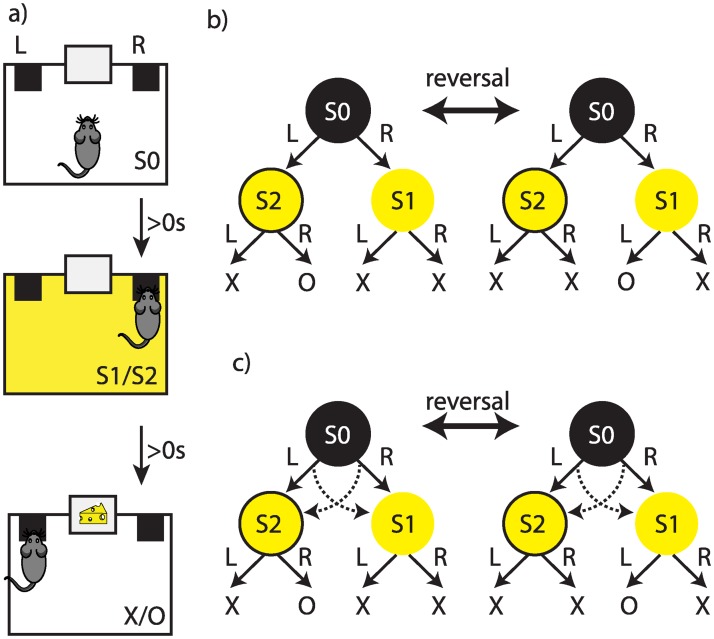
(a) The flow of events in the two-stage task. Trials started in state S0, which was signalled by the absence of the house light. After an action (‘L’ or ‘R’) was taken at stage 1, either constant or blinking house light started (S1 or S2). Next, subjects could take another action at stage 2 (‘L’ or ‘R’), which could lead either to the delivery of the outcome or to no outcome. Actions taken in S0 immediately lead to the presentation of either S1 or S2, and actions taken in S1 or S2 immediately lead to the outcome or no outcome. The inter-trial interval (ITI) was zero in this experiment, but in the experiments reported in the S5, S6 and S7 Figs it was greater than zero, as detailed in S2 Text. (b) The structure of the task. Stage 1 actions in S0 led to the stage 2 stimuli (S1/S2) in a deterministic manner. The rewarding stage 2 state changed with a probability of 0.14 after earning an outcome (indicated by ‘reversal’ in the graph). ‘O’ represents outcome, and ‘X’ no-outcome. (c) The structure of the probe sessions. The probe sessions were similar to the training sessions (panel (b)), except that stage 1 actions led to the stage 2 states in a probabilistic manner. Taking action ‘L’ led to state S2 commonly (80% of the time), and to state S1 rarely (dashed lines). Taking action ‘R’ led to state S1 commonly (80% of the time), and to state S2 rarely (dashed lines).

Rats (n = 8; see Material and methods) received several pre-training sessions, in which they learned to press two levers, ‘R’ and ‘L’ (right and left), to earn food pellets (Fig 1:phase 2). Subjects then learned to discriminate between two stimuli S1 and S2 such that in the presence of S2 action ‘R’ was rewarded whereas in the presence of S1 action ‘L’ was rewarded (Fig 1:phase 3; S4 Fig). Subsequently, the rats received training on a two-stage decision-making task, in which they first made a binary choice at stage 1 (S0), after which they transitioned to one of the stage 2 states, (either S1 or S2), in which again they made another binary choice that could lead to either reward delivery or no-reward (Fig 2a). In each trial, only one of the stage 2 states led to reward; the other state did not lead to reward irrespective of the choice of actions (Fig 2b). During the course of each session, the stage 2 state that earned a reward switched without any signal (with probability 0.14; indicated by ‘reversal’ in Fig 2b).

### Adaptive state-space representation

**Fig 3:**
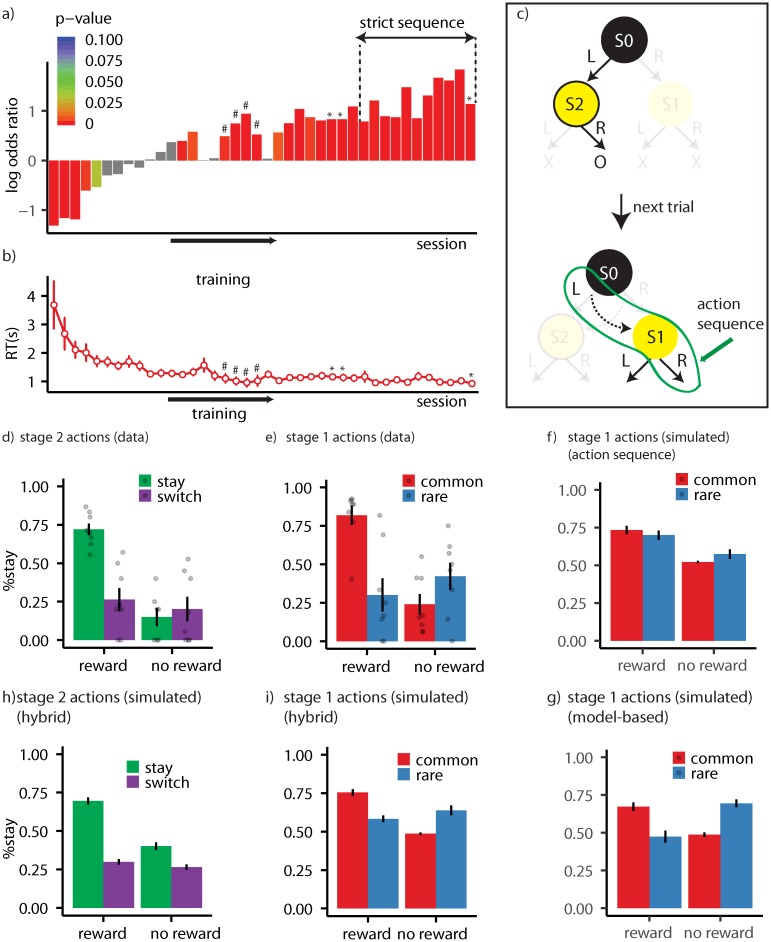
(a) Logarithm of odds ratio of staying on the same stage 1 action after getting rewarded on the previous trial over the odds ratio after not getting rewarded. The zero point on the *y*-axis represents the indifference point (equal probability of staying on the same stage 1 action after reward or no reward). Each bar represents the odds ratio for a single training session. In the sessions marked with ‘#’ in Figure 3a the contingency between stage 1 actions and stage 2 states were revered (‘L’ leads to S1 and ‘R’ to S2). ‘Strict sequence’ refers to sessions in which a trial was aborted if the animal entered the magazine between stage 1 and stage 2 actions. Sessions marked with ‘*’ are probe sessions in which the task involved both rare and common transitions. (b) Reaction times (RT) averaged over subjects. RT refers to the delay between performing the stage 1 and stage 2 actions. Each dot represents a training session. (c) An example of how the performance of action sequences can be detected in the probe session. On a certain trial a rat has earned a reward by taking ‘L’ at stage 1 and ‘R’ at stage 2. The subject then repeats the whole action sequence (‘L’ and then ‘R’), even though after executing ‘L’ it ends up in S1 (due to a rare transition) and action ‘R’ is never rewarded in that state. (d) The probability of staying on the same stage 2 action in the probe session averaged over subjects, as a function of whether the previous trial was rewarded (reward/no reward) and whether subjects stayed on the same stage 1 action (stay/switch). As shown in panel (c) only the trials in which state 2 state is different from the previous trial are included. (e) The probability of staying on the same stage 1 action in the probe session averaged over subjects as a function of whether the previous trial was rewarded (reward/no reward) and whether the transition in the previous trial was common or rare. (f) Model simulations depicting the probability of staying on the same stage 1 action when the model is using exclusively action sequences. (g) Model simulations depicting the probability of staying on the same stage 1 action when the model is using the true state-space of the task but not action sequences. (h) Simulation of stage 2 choices, and (i) stage 1 choices using the best-fitted parameters for each subject. Error bars represent ±1 SEM.

The stage 2 state that earned reward changed over time and, as such, subjects needed to use feedback from the previous trial to track which specific stage 2 state was rewarded so as to take the stage 1 action leading to that state. Given this situation, it should be expected that, if a reward is earned on the previous trial, the subjects will repeat the same stage 1 action on the next trial. Fig 3a shows the logarithm of odds ratio of staying on the same stage 1 action after earning a reward on the previous trial over the odds after earning no reward (across training sessions). Each bar in the graph represents a training session, and odds ratios were calculated using logistic regression analysis on the effect the reward had on staying on the same stage 1 action on the next trial (see Material and methods for details). The zero point on the *y*-axis in the figure shows the indifference point, i.e., when the probability of staying on the same stage 1 action after earning reward or no reward is equal. As the figure shows, in early training sessions the rats failed to show a tendency to take the same stage 1 action after earning a reward on the previous trial and instead showed a tendency to switch to the other action (first five sessions; *β* = −0.929 (CI: −1.194, −0.664), SE = 0.135, *p* < 10^−11^). This sub-optimal behaviour can be explained with reference to the state-space and action representation that decisions were based on early in training; i.e., subjects were initially unaware that the environment had two stages, treated it as a single stage environment, and therefore repeated the action taken immediately prior to reward delivery. For example, if they took ‘L’ at stage 1, and ‘R’ at stage 2 and earned reward, they repeated action ‘R’ at the beginning of the next trial. Although this looks like they switched to the other stage 1 action, they were clearly repeating the last rewarded stage 2 action, which should be expected if decisions are made as if the environment is composed of a single stage.

Therefore, actions were not based on a two-stage representation, which would require that the subjects treat S1 and S2 as the outcomes of actions taken in S0; indeed, to the contrary, the data shows that the subjects acted as if their next action in S0 led to reward directly. This could be for two reasons: (1) Although S0 was visually distinct from S1 and S2, early in training it may not yet have been part of the state-space and therefore the outcomes of actions taken in S0 (which are S1/S2) were not differentiated from the outcomes of the actions taken in S1/S2 (which were reward/no-reward). From this perspective, a reward earned by taking an action in S1 or S2 was attributed to taking actions in the upcoming S0 and led the rats to repeat the same action in S0. Alternatively, (2) S0 was part of the state-space and was being treated differently from S1/S2, but the rats had yet to encode that ‘L’ leads to S2 and ‘R’ to S1. The former hypothesis relates to the state-space representation, whereas the latter relates to learning the “transition matrix” of the task, i.e., state-action-state relationships. There are two points in favour of the first hypothesis. Firstly, if animals were confused about what will happen after taking stage 1 actions (i.e., whether they will lead to S1 or S2), then we would expect the subjects to act randomly in S0, whereas, as the data indicate, the subject repeated the last rewarded action in S0 as if S0 was similar to the state in which the last action was taken. Secondly, animals typically learn action-outcome contingencies very rapidly. For example, in a simple instrumental conditioning experiment in which two levers lead to different (motivationally relevant) outcomes, such a food pellets, animals are able to learn the contingencies in a single training session [14], whereas here it took animals more than ten training sessions to take the correct actions in S0. Based on this finding, we interpret the effect as a consequence of the rats forming a simple state-space representation, i.e., all the states are organised into a single stage early in training, which is shown as S0/S1/S2 in Table 1a.

**Table 1:** Four hypotheses about state-space and action representations. (a) The state-space constitutes a single stage (outcomes such as food pellets are not shown here as states), and ‘L’ and ‘R’ are the only possible actions that the subject considers taking. (b) The state-space matches the correct state-space of the task, and the actions are ‘L’ and ‘R’. (c) The state-space only consists of state S0, and actions are ‘L → R’ and ‘R → L’. Note that single actions ‘R’ and ‘L’ are not included. (d) The state-space represents the two stages of the task and actions include both single actions and action sequences.

	(a)	(b)	(c)	(d)
state-space	S0/S1/S2	S0, S1, S2	S0	S0, S1, S2
actions	L, R	L, R	L→R, R→L	L, R, L→R, R→L

A final logically possible account can be formed based on the assumption that S0 is part of the state-space but S1 and S2 are not. This account can explain why animals do not treat S1 and S2 as the outcome of S0, but is inconsistent with the fact that the rats took different actions in these states and so were clearly able to discriminate between S1 and S2.

Importantly, as Fig 3a shows, this pattern of choices reversed as the training progressed and the rats started to take the same stage 1 action that earned reward on the previous trial rather than repeating the action most proximal to reward (see S3 Fig for the behaviour of individual subject). Replications of this finding using other experimental parameters are provided in Supplementary Experiments 1-3 in (S5, S6 and S7 Figs and S2 Text). One explanation for this observation is that, at this point in training, the rats realised that the task had two-stages and, at that point, acquired the “correct” state-space of the task (Table 1b), where the correct representation refers to the Markov Decision Process underlying the task structure [note that defining the correct state-space is not trivial; see 15]. If this is true, however, then, during the course of training, the state-space used by the animals expanded from a simple representation (Table 1a) to a more complex representation consistent with the task state-space (Table 1b).

### Adaptive action representation

Learning the state-space of the task is not the only way that the rats could have adapted to the two-stage structure of the environment; in this task reward can be earned either by executing ‘L’ at stage 1 and ‘R’ at stage 2, or by executing ‘R’ at stage 1 and ‘L’ at stage 2. As such, it is possible that animals chunked actions ‘L’ and ‘R’ to make action sequences; say, ‘L→R’, and ‘R→L’. Using these new actions, the rats could then repeat an action sequence on the next trial after earning a reward instead of merely repeating the action proximal to the reward, as early in training. If this is true, however, then the transition in the pattern of stage 1 actions shown in Fig 3a could have been driven by the rats acquiring action sequences rather then the state-space of the task. This assumption is also consistent with the fact that the delay between the first and second action (the rats’ reaction time) decreased as training progressed (Fig 3b), consistent with the formation of action chunks.

In order to test the role of action sequences, we looked at the choices of the subjects in a *probe session* inserted at the end of the training sessions (last bar in Fig 3a), in which in a small portion of the trials transitioning between stage 1 actions and stage 2 states were switched (Fig 2c). For example, during training (non-probe sessions), after executing action ‘L’ subjects always ended up in state S2; however, the probe session included some rare transitions (20% of the trials), in which, after taking ‘L’, subjects ended up in state S1 instead of S2 [inspired by the two-stage task in 9]. As a consequence, in the probe session, after repeating the same stage 1 action subjects might end up in a different stage 2 state than on the previous trial, meaning that they would next take a different stage 2 action if they are selecting actions one by one. If, however, subjects are repeating the previously rewarded action sequence, we should expect them to repeat not only the first action, but also the second action even if they are now in a different stage 2 state [16, 6, 10]. Fig 3c shows an example of this situation. Animals have earned reward from action sequence ‘L→R’ on the previous trial and have repeated action ‘L’ at stage 1 of the next trial, but on this trial have ended up in state S1 in which action ‘L’ should be taken. If, however, they take action ‘R’ this can be taken as a sign that they are repeating the whole action sequence rewarded on the previous trial.

Fig 3d shows the probability of staying on the same stage 2 action on the trials in which the stage 2 state is different from that of the previous trial. As the figure shows, if the previous trial is rewarded (‘reward’ condition) and subjects stay on the same stage 1 action (‘stay’ condition) then there is a high chance that they will also repeat the same stage 2 action, indicating that they are repeating the whole previously rewarded action sequence. This is supported by a significant interaction between staying on the same stage 1 action and reward on the previous trial (*β* = 0.494 (CI: 0.055, 0.933), SE = 0.224, *p* = 0.027; see Table 2:stage 2 for the full analysis). Therefore, the pattern of choices at stage 2 is consistent with the suggestion that the subjects have expanded the initial set of actions, that previously only included actions ‘L’ and ‘R’ (Table 1a), to a more complex set that includes action sequences ‘L→R’ and ‘R→L’ (Table 1c).

**Table 2:** Results of the logistic regression analysis of stage 1 and stage 2 choices in the probe session. For the stage 1 choices, the analysis focused on staying on the same stage 1 action on the next trial, based on whether the previous trial was rewarded and whether it was common or rare (trans). ‘reward:transition’ is the interaction between reward, and transition type. For stage 2 choices, the analysis focused on staying on the same stage 2 action, based on staying on the same stage 1 action (stay) and earning a reward on the previous trial. ‘reward:stay’ is the interaction between ‘reward’, and ‘stay’.

**stage 1 actions**				
	intercept	reward	transition	reward:transition
*p*-value	0.373	0.003	0.164	< 10^−5^
*β* (SE)	-0.16 (0.18)	0.61 (0.20)	0.37 (0.26)	0.92 (0.20)
**stage 2 actions**				
	intercept	reward	stay	reward:stay
*p*-value	0.001	<0.001	0.019	0.027
*β* (SE)	-0.95 (0.29)	0.84 (0.23)	0.60 (0.25)	0.49 (0.22)

It is important to note that the emergence of such actions sequences depended on the experimental setting; as reported in the S5, S6 and S7 Figs and S2 Text, we did not find evidence of action sequences using particular experimental parameters; see Section ‘Choice of experimental parameters’ below.

### The integration of adaptive state-space and action representations

The analysis provided in the previous section showed that acquiring either the state-space representation or action sequences can explain the pattern of choices observed during the course of training (Fig 3a). Furthermore, the pattern of choices at stage 2 of the probe session provided evidence that the subjects are using action sequences. It remains open to question, therefore, whether the rats are exclusively solving the task using action sequences without relying on the state-space of the task (Table 1c), or are using both an expanded state-space and action representations for decision-making (Table 1d). To answer this question we looked at the pattern of choices at stage 1 of the probe session.

As argued in the previous sections, if decisions are based on the true state-space of the task then we expect that, after earning reward on a trial, the same stage 1 action will be taken on the next trial. The same is not true for the trials with rare transitions in the probe session, however. This is because, if the reward was earned on a trial with a rare transition, the subjects should then switch to the other stage 1 action on the next trial if they are using their knowledge of the state-space of the task [9]. For example, imagine it is a trial with a rare transition and the rat, by taking ‘L’, is transferred to state S1 and earns reward. On the next trial, using the state-space of the task, the rat should switch to ‘R’ at stage 1 because ‘R’ is the stage 1 action that commonly (80% of time) leads to S1. As a consequence, staying on the same stage 1 action after earning reward depends both on the reward and the transition type on the previous trial.

On the other hand, if the rats are exclusively using action sequences without relying on the state-space of the task (Table 1c), then staying on the same stage 1 action only requires that the previous trial was rewarded; the transition type of the previous trial should not have any effect. This is because earning reward by executing an action sequence will result in the same action sequence being repeated on the next trial (and so the same stage 1 action) irrespective of the transition type on the previous trial. Therefore, a main effect of reward on staying on the same stage 1 action in the next trial indicates that the subjects are using action sequences (Fig 3f) whereas, an interaction between reward and transition type on the previous trial indicates that the subjects are using the true state-space of the task (Fig 3g).

Importantly, the results of stage 1 actions, presented in Fig 3e, clearly revealed a significant reward-transition interaction (Table 2:stage 1), indicating that the subjects were using the correct state-space of the task (Table 1b). In addition, the main effect of reward was also significant, which indicates that the subjects were also using action sequences (Table 1c). As such the pattern of choices indicates that the rats were using both action sequences and single actions guided by the true state-space of the task. Therefore, evidence from this study suggests that, as training progressed, the initially simple state-space and action representations (Table 2a) were expanded to align with the true structure of the task (Table 2d).

Note that there are other explanations for the main effect of reward, other than using action sequences. For example, it could be the case that after experiencing a rare transition, subjects presumed that the relationship between stage 1 actions and stage 2 states had switched, which predicts a main effect of reward on staying on the same stage 1 action even if subjects were not using action sequences. Another explanation for the main effect of reward is based on the notion of ‘model-free’ actions. Intuitively, this implies that earning reward after taking an action increases the chance of repeating the action; i.e., on this task, that reward increased the chance of taking the same stage 1 action on the next trial whether the experienced transition was common or rare [9]. Nevertheless, although these two accounts can predict a main effect of reward, they do not predict nor can they explain the effect observed on the stage 2 actions (as explained above in Section Adaptive action representation).

As mentioned earlier, we did not find evidence for the operation of action sequences in all the range of experimental parameters that we tested and the probe tests that we conducted (as described below in section ‘Choice of experimental parameters’). For the conditions that we did not find evidence for the operation of action sequences, the main effect of reward mentioned above can be attributed either to the model-free effects—as if animals did not acquire action sequences at all [9]–, or alternatively to the interruption of actions sequences, i.e., animals started action sequences, but because of the experimental parameters they managed to inhibit the execution of inappropriate sequences. Our results cannot distinguish between these two explanations, a point that we will expand on in the Discussion section.

### Potential effects of latent states and action biases

In the previous section, we argued that the reward-transition interaction is a sign the animals had acquired an adaptive state-space representation, which allowed them to learn the relationship between stage 1 actions and stage 2 states. However, recently, [17] argued that this form of reward-transition interaction in multistage decision-making can be explained if subjects have learned the ‘latent states’ of the task without relying on the relationship between stage 1 actions and stage 2 states. On this account, the rats simply learned a kind of rule: e.g., whenever a reward is earned in S1, perform ‘R’ on the next trial (at stage 1), and whenever a reward is earned in S2, perform ‘L’ on the next trial. [17] argue that this process requires the subjects to expand their representation of the state-space by turning S0 into latent states S0S1 and S0S2, which encode which stage 2 state was rewarded on the previous trial and, therefore, their argument depends on the rats expanding their representation of the state-space to include new states. As a consequence, even under [17]’s account, the observed reward-transition interaction is evidence for an adaptive state-space representation, as we have argued. Furthermore, this account cannot explain the adaptive action representations that we observe; i.e., the pattern of choices due to the formation of action sequences. This is because [17]’s account does not imply repeating the previously rewarded sequence of actions in S0 and S1/S2.

There is another potential interpretation of the reward-transition interaction based on the potential for a local response bias induced by the reward function. Assume that, in a part of the probe session, actions taken in S1 are rewarded (and actions taken in S2 are not), and by trial and error the animal develops a tendency to take action ‘R’ more frequently than ‘L’ at S0; i.e., the probability of staying on the same action when it is ‘R’ is higher than when it is ‘L’. As most of the common transitions after taking ‘R’ are rewarded (as they mostly lead to S1) and most of the rare transitions are non-rewarded (as they mostly lead to S2), there will be an effect of reward-transition interaction on the probability of staying on the same action, which looks like the animals are taking the structure of the world into account, while what they are doing is simply taking action ‘R’ more frequently. This issue was discussed in [10, 18] and further analysed in [17] and one way to address it is to add a new predictor to the analysis of the effect of reward and transition on staying on the same stage 1 action. This new predictor encodes whether the previous stage 1 action was the best action, i.e., it leads to the stage 2 state with the highest reward, which will absorb the effect of the reward-transition interaction if the interaction is just due to repeating the best action more frequently [18, 17]. This analysis is presented in S5 Table, which shows that even in the presence of this predictor the effect of reward and the reward-transition interaction are still significant. As such, the reward-transition interaction is unlikely to be due to this form of response bias.

### Choice of experimental parameters

Animals were given three probe sessions in total, and the results reported above were taken from the last of these tests which was the final experimental session (probe sessions are marked by an asterisk in Fig 3a). The full analysis of all the probe sessions is presented in see S4 Table. The structure of the probe sessions was identical to each other and also in terms of results; similar to the third probe session analysed above, the main effect of reward was significant in sessions one and two. However, unlike the last probe session, in the first two sessions, the rats did not show evidence that they were using action sequences (S4 Table: probe 1 and 2, stage 2 actions; reward-stay interaction; *p*-value>0.1). A closer examination of these sessions revealed that, at this stage in the training, the rats were not discriminating between the stage 2 stimuli; i.e., because the analysis of stage 2 only included trials in which the stage 2 state is different from the previous trial, we expected the probability of staying on the same stage 2 action to be generally low (as different actions are rewarded in the stage 2 states), which was not the case in the first two probe sessions (see S4 Table; *p*-value > 0.05 for the intercept term at stage 2 actions in probe 1, 2). As a consequence, under these conditions, staying on the wrong stage 2 action due to the performance of action sequences cannot be detected, because the rats are likely to take the incorrect action at stage 2 states even if they are not taking an action sequence (ceiling effect). One reason for this lack of discrimination is the potential for interference between the stage 1 and stage 2 actions; if the rats checked the magazine after taking the stage 1 action they may then have repeated the same stage 1 action instead of taking the correct stage 2 action. This would make it look like the animals were not discriminating between stage 2 stimuli. To address this issue we introduced the ‘strict sequences’ criterion for the next ten training sessions (Fig 3e) under which a trial was aborted if a rat entered the magazine between the stage 1 and stage 2 actions. After these ten training sessions the rats were given the third probe test, in which they showed they were able significantly to discriminate between the stage 2 states (see Table 2; *p*-value = 0.001 for the intercept term at stage 2 actions). Note that the analysis presented in the previous and subsequent sections relates to this last probe session.

In S5, S6 and S7 Figs, S2 Text we also present three supplemental experiments each of which used different parameters. Supplementary experiment 1, which is shown in S5 Fig, includes probe sessions throughout the training process and shows the development of choices. Supplementary experiments 2 and 3 are mostly similar to each other and provide a training protocol in which animals reliably exhibit the reward effect in their stage 1 actions. However, in none of these experiments were we able to observe the performance of action sequences, as indicated by the reward-same interaction in stage 2 actions (see S6 Table for the full analysis of supplementary experiment 1 and S8 Table for the full analysis of supplementary experiments 2,3). One main difference between these experiments and the experiment reported in the main paper is that, whereas in the main experiment the ITI was zero, in the supplementary experiments, the inter-trial interval was non-zero. In this latter condition, animals were often found to take actions during the ITI, which were not rewarded but which were very likely to interfere with the performance of action sequences once the next trial started. Using an ITI of zero addressed this issue. Lastly, as Fig 3a shows, there were some training sessions in which the contingency between stage 1 actions and stage 2 states was reversed. These training sessions were introduced to overcome the interference that could be produced when animals were first exposed to rare trails.

### Computational models of adaptive decision-making

We next sought to establish the computational model that best characterized the decision-making process used by the rats in this experiment. The modelling was focused on the probe session that we analysed in the previous sections (the final probe session). For this purpose, we compared different families of reinforcement-learning (RL) model to establish which provided a better explanation for the data (Table 3). The families compared included: (1) a non-hierarchical model-based RL family (MB) corresponding to Table 1b, which assumes that the subjects acquired the correct state-space of the task, but in which action sequences were *not* included in the set of actions; (2) a hierarchical RL family (H) corresponding to Table 1c, which assumes that the set of actions included only action sequences, but that decisions were *not* guided by the true state-space of the task; (3) a hierarchical model-based RL family (H-MB) corresponding to Table 1d, which assumes that the subjects were using single actions, action sequences and the true state-space representation for decision-making [10]; (4) a model-free RL family (MF) without action sequences but using the correct state-space (5) a model-free RL family with action sequences and single actions and correct stat-space of the task (H-MF), (6) a hybrid model-based RL and model-free RL family with only single actions and without action sequences using the correct state-space (MB-MF), and (7) a hybrid of model-based and model-free RL with both action sequences and single actions (H-MB-MF). Model-free RL has been previously used to characterise performance on a similar task [9], and here we used it as a baselines in mixture with model-based RL and hierarchical accounts.

**Table 3:** States and actions for each family of computational model along with degrees-of-freedom (df), pseudo-*r*^2^, and negative log model-evidence (− log *p*(*D*|*M*)) for the best model in each family; lower values of negative log model-evidence are better.

family	state-space	actions	df	p-*r*^2^	negative log model-evidence
MB	S0, S1, S2	L, R	4	0.175	1222.576
H	S0	L → R, R → L	5	0.207	1185.274
H-MB	S0, S1, S2	L, R, R → L, L → R	4	0.207	1172.357
MF	S0, S1, S2	L, R	4	0.175	1219.870
MB-MF	S0, S1, S2	L, R	5	0.180	1223.515
H-MF	S0, S1, S2	L, R, R → L, L → R	4	0.204	1179.717
H-MB-MF	S0, S1, S2	L, R, R → L, L → R	5	0.211	1178.339

**Fig 4:**
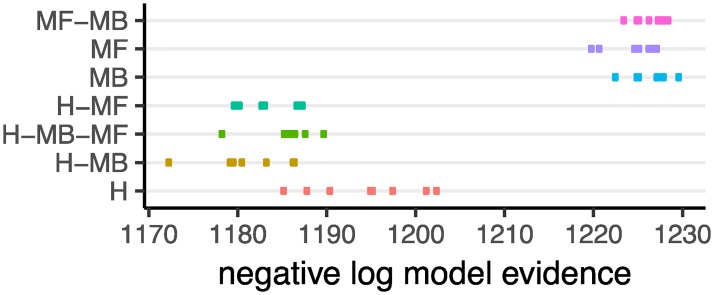
Negative log model-evidence (− log *p*(*D*|*M*); lower numbers indicate better models) for the first best eight models in each family of computational models. Different models are shown on the y-axis using different colours for better visualisation.

In total we considered 536 different models in which each family consisted of several members with different degrees of freedom (see S1 Text for details). We then calculated the negative log model-evidence for each model *M* given the choices of subjects, *D* (denoted by − log *p*(*D*|*M*)). Table 3 shows the negative log model-evidence along with other properties of the best model for each family. The differences in log model-evidence (log-Bayes factor) between the best fitting model of the H-MB family and other families was greater than 5. In the Bayesian model comparison literature, log-Bayes factors greater than 3 are considered to be strong evidence [19]. Therefore, the above results provide strong evidence that the subjects were utilising H-MB to guide action selection compared to the other models. Fig 4 shows the negative log model-evidence for the best eight models in each family and shows that the best model in the H-MB family provides a better explanation of the data than any of the other families.

We then simulated eight instances of the H-MB model of the task using the best fitting parameters for each subject (S2 Table) and analysed the stage 1 and stage 2 choices of the simulated model. Analysis of stage 1 choices (Fig 3i) revealed a significant main effect of reward (*β* = 0.293 (CI: 0.217, 0.369), SE = 0.038, *p* < 0.001), and a significant interaction between whether the previous trial was rewarded and the transition type of the previous trial (*β* = 0.327 (CI: 0.233, 0.422), SE = 0.048, *p* < 0.001). Analysis of stage 2 choices (Fig 3h), revealed a significant interaction between earning a reward on the previous trial and the likelihood of staying on the same stage 1 action (*β* = 0.209 (CI: 0.091, 0.327), SE = 0.060, *p* < 0.001). These results are, therefore, consistent with the behavioural results of our experiments using rats as subjects. The fact that the H-MB family provides a better fit than the H family implies that subjects were using the correct state-space of the task (the MB part), whereas the finding that the H-MB family were better than the MB family implies that subjects were using action sequences. See S8 Fig for the similar simulations using the best fitting model in other classes of models. See S1 Table for the negative log-model evidence of the best model in each family.

The H-MB family also provided a better fit than baseline MB/MF models, however, this does not imply that some form of model-free RL is not working concurrently with a H-MB model. Indeed, one can imagine a model which includes both MB and MF components operating hierarchically over action sequences. Whether such a model provides a better fit of the data than H-MB cannot be addressed using the current task because it will require manipulating the value of the outcomes. The conclusions made earlier about learning the state-space and action sequences are orthogonal to the role that MF RL plays in these decisions, and therefore they are not affected by this limitation.

## Discussion

Learning the value of different actions in various states of the environment is essential for decision-making in multi-stage environments. This learning process operates above the state-space and action representation and, therefore, the ability to (i) acquire the correct state-space of the task, and (ii) create new actions that are useful for solving the task, are important for efficient decision-making. Using a sequential decision-making task in rats, we provide direct evidence that, early in training, subjects make decisions based on simple state-space and action representations, but that, during the course of training, both state-space and action representations (under certain conditions) evolve and adapt to the structure of the environment. That is, we found that the rats responded initially as if the proximal response to reward was the only relevant action to earn that reward but gradually learned the interaction between the first lever press and the second lever press response within and across the states signalled by the discriminative stimuli and so acquired the multistage discrimination. Furthermore, we found evidence that the single lever press actions initially acquired by the rats later expanded to include sequences of lever presses and that, when these sequences were used, they tended to be used in a habitual manner by repeating previously rewarded sequences even when the stage 2 state was revalued.

The ability to solve multi-stage decision-making tasks has been previously demonstrated in different species, however, unlike the current study, these demonstrations have either given the different stages of the task to the subjects [e.g., 9], or have explicitly signalled the actions that should be taken at each stage [e.g., 20], which remove the necessity for building multi-stage representations to solve the task. Similarly, the ability of animals to concatenate simple actions to execute action sequences has been established previously and here we extended these prior studies by showing that, during the course of learning, single actions turn into action sequences that are not only executed, but also are evaluated as a single response unit [13]. Similar findings have recently been reported demonstrating that lever press-magazine entry sequences are resistant to devaluation in rats [21].

A task similar to the two-stage task that we used here in rats has previously been employed to study different forms of decision-making processes in humans [9]. Although the experiments in those studies were composed of a single session, results indicated that the subjects were using an expanded state-space representation without needing to go through multiple training sessions. This is presumably due to the instructions and the cover story provided to the subjects, which informed them about the correct state-space representation. In terms of acquiring action sequences, using a similar task in humans we have previously shown that subjects engaged action sequences [10]. Again, however, we found they were able to do so without requiring multiple training sessions. Why such sequences should have formed so rapidly is a matter of conjecture but, as the task involved pressing keys on a keyboard, familiarity with similar response sequences could have supported sequence acquisition (especially as only two key presses were required to earn the reward). Based on these comparisons, the results of the current experiments point to the importance and complexity of learning state-space and action representations. As the profile of the rats’ choices indicates, they required a significant amount of training in order to learn the structure of the environment (10-40 sessions). This is while, in some instrumental conditioning settings, animals are able to learn the contingency between actions and states in two and sometimes in a single training session [14]. Uncovering the processes that determine the encoding of the state-space of the task and how this process interacts with that subserving instrumental conditioning will be an important step towards better understanding the learning mechanisms that mediate decision-making processes generally.

The results of the computational modelling indicated that hierarchical model-based RL provides the best explanation for the rats’ choices. This model assumes that the subjects build an internal map of the environment which encodes both the specific outcomes of single actions *and* of action sequences. The validity of this assumption for single actions can be confirmed based on the results of the current experiment and previous studies [22] showing that subjects encode the specific outcome of each individual action, e.g., taking ‘R’ leads to ‘S1’ and ‘L’ leads to ‘S2’. With regard to encoding the outcome of action sequences, although previous studies have indicated that the subjects specifically encode the outcome of each action sequence [13], we cannot assess whether subjects encoded outcome specific sequences in the current study because the value of the food outcome was not manipulated. As such, the results do not address the (model-based or model-free) nature of the controller mediating the evaluation of action sequences.

Although the computational modelling analysis provided evidence consistent with hierarchical model-based RL, the results do not imply that all the behavioural trends in the data were captured by the model. In particular, although a wide range of models with different parameters were considered here, there were some differences between the pattern of stage 1 choices in the data shown in Fig 3(e) and the simulations of the best model shown in Fig 3(i). One way to address this issue is using recurrent neural networks (RNNs) instead of the RL family, which are more flexible and able to learn the details of the behavioural processes without relying on manually engineering the models [23]. Another limitation of the current work is that, although we provided evidence for the expansion of the state-space of the task, we did not provide any computational account for ‘how’ the states-space is acquired by the animals. This can potentially be addressed by approximating *Q*–values using a parametrised family of models able to adjust their predictions as the training progresses.

Along the same lines, as we discussed in the previous sections, the emergence and detection of action sequences requires certain experimental conditions, such as a short ITI. Without these conditions actions at stage 1 are consistent with the operation of action sequences, but not actions at stage 2. One explanation for this effect is the possibility that action sequences were inhibited. For example, if during long ITIs the subjects go through extinction, it is unlikely that they keep performing the whole action sequence throughout the ITI and into the next trial. As such, although the first component of the action sequence at stage 1 is performed, the second component may be inhibited when it is inappropriate, making it harder to detect the performance of action sequences. Alternatively, this pattern of choices could also indicate the operation of another RL system, such as model-free RL, instead of interrupted sequences. Within this alternative framework, choices are a mixture of goal-directed actions (model-based), and model-free actions that are guided by their ‘cached’ [as opposed to their current values]. Our results are equivocal with respect to this interpretation, but since, in other conditions, there is positive evidence for action sequences, it is more parsimonious to interpret this result in terms of the inhibition of action sequences rather than as the output of an additional model-free system.

The current task and the parameter set that we have assessed in these experiments (see also S2 Text) constitute an addition to the developing literature on multistage discrimination learning [e.g., 17, 12, 11, 24, 25] and its relationship to action sequence learning in rats [13]. Importantly, this research demonstrates that one way in which rats, and potentially other animals including humans [10], learn complex multistage discriminations is not just by expanding the task space through shifts in attention across the perceptual feature dimensions of complex stimuli [26, 27] but also by expanding the action repertoire from single actions to action sequences. The tendency to generate such sequences may have been aided by some important features of the current task; perhaps the most important of which was our attempt to minimize the impact of predictive cues in the first stage that could significantly interfere with the action-related predictions of the second stage state. However, a recent study [28] that replicated many of the effects reported by [10] with regard to the development of action sequences in a multistage discrimination task in human subjects nevertheless confounded stimulus and action predictions at the first stage as others have done [9]. However, the Adams and Cushman’s study differed from Dezfouli and Balleine’s in also reporting some evidence for a model-free component of performance over and above the model-based selection of habitual action sequences, which raises the possibility that evidence of model-free RL in first stage choices depended on using specific first stage stimuli that could provide stimulus values for the second stage states. That feature aside, however, the current task is a close analogue for the human 2 stage task generated by Daw and colleagues and particularly that of [10] and appears to produce very similar effects in both rodent and human subjects.

Given its utility in animals, this task could, therefore, provide the opportunity to not only investigate the behavioural and psychological determinants of performance across species but, by utilising more direct neural manipulations, to establish the way in which the brain supports state and action learning in multistage discrimination tasks and particularly how model-based control of simple actions and habitual actions sequences is implemented in multistage discriminations. Generally, the interaction of a model-free controller with action sequence learning has not been evaluated at the neural level, although considerable evidence suggests that premotor and presupplementary motor regions are likely to play an important role [e.g., 29]. This is particularly true given the evidence in animals that damage to the rodent homologue of premotor cortex reduces otherwise sequential action-outcome relations simple actions [13]. This account is strikingly different from other models within which goal-directed actions and habits and their computational model-based and model-free implementation have typically been seen as antagonistic processes for which some form of arbitrator is required to adjudicate competition between them. Arbitration has been investigated in a number of paradigms in humans and several investigators have found evidence of model-based model-free arbitration-related activity particularly in the inferior lateral prefrontal cortex [30, 31]. In contrast, within hierarchical models, goal-directed actions and habit sequences do not compete, being two action strategies from which the goal-directed controller selects. Which of these is the more accurate statement of the way goal-directed and habitual actions interact is an empirical issue but other potential models of performance have generally suggested that collaboration rather than competition between action controllers more accurately captures their interactions [cf. 32].

## Methods and materials

### Subjects and apparatus

Eight experimentally naive male Hooded Wistar rats served as subjects in this study. Data from all the subjects are included in the analyses. All animals were housed in groups of two or three and handled daily for one week before training. Training and testing took place in eight Med Associates operant chambers housed within sound- and light-resistant shells. The chambers were also equipped with a pellet dispenser that delivered one 45 mg pellet when activated (Bio-Serve). The chambers contained two retractable levers that could be inserted to the left and the right of the magazine. The chambers contained a white noise generator, a Sonalert that delivered a 3 kHz tone, and a solenoid that, when activated, delivered a 5 Hz clicker stimulus. All stimuli were adjusted to 80 dB in the presence of a background noise of 60 dB provided by a ventilation fan. A 3 W, 24 V house light mounted on the wall opposite the levers and magazine illuminated the chamber. Microcomputers equipped with MED-PC software (Med Associates) controlled the equipment and recorded responses. Animals were food deprived one week before starting behavioral procedures. They were fed sufficiently to maintain their weight at 90% of their free-feeding weight. The animals were fed after the training sessions each day and had free access to tap water whilst in their home cage. Each training session (except the magazine training sessions) started with insertion of the levers, and ended with their retraction. All procedures were approved by the University of Sydney Animal Ethics Committee.

### Behavioral procedures

Rats were given two sessions of magazine training in which 30 grain pellets were delivered on a random time 60-s schedule (Fig 1:phase 1). Rats were then trained to lever press on a continuous reinforcement schedule with one session on the left lever and one session on the right lever each day for four days with the total number of outcomes each day limited to 60 per session (Fig 1). The total duration of each session was limited to 60 minutes (see S2 Fig for the average session duration). Next, rats were trained to discriminate the two stimuli (Fig 1:phase 3). Each session started with the presentation of a stimulus. The stimulus was presented until the rat performed an action (either pressing the left or right lever) after which the stimulus turned off. For one stimulus, taking the left action led to the reward, whereas for the other stimulus taking the right action led to reward. Levers and stimuli were counterbalanced across subjects. After an action was chosen, there was a 60-second inter-trial interval (ITI) after which the next trial started with the presentation of the next stimulus, again chosen randomly. The duration of each session was 90 minutes, with no limit on the maximum number of earned rewards. The stimuli were a constant or a blinking house light (5 Hz). The result of this phase is depicted in S4 Fig.

The rats then received training on the two-stage task depicted in Fig 2b (maximum 60 outcomes in a session and maximum duration of a session was limited to 1 hour). Animals were trained on the two-stage task for 40 sessions. In the middle of, or at the end of these training sessions, they were given probe sessions, similar to the training sessions except that stage 1 actions led to stage 2 states in a probabilistic manner (Fig 2c). These sessions are indicated by ‘*’ in Fig 3a. After the first two training sessions, subjects then received ten more training sessions, and were then given a further probe test. The results reported in the Results section correspond to this last probe session. In the sessions marked with ‘#’ in Fig 3a the contingency between stage 1 actions and stage 2 states were reversed (‘L’ leads to S1 and ‘R’ to S2); these training sessions were followed by a session in which ‘L’ leads to S1 and ‘R’ to S2 in 20% of times, followed by normal training sessions, as Fig 3a shows. Finally, ‘strict sequence’ in Fig 3a refers to a session in which a trial was aborted if the animal entered the magazine between stage 1 and stage 2 actions. In all the training phases levers were present throughout the training session. See S3 Table for the total number of trials completed by each subject; see S7 and S9 Tables for the total number of trials completed by subject in the supplementary experiments.

### Behavioral analysis

We used R [33] and lme4 packages [34] to perform a generalized linear mixed effects analysis. In all of the analyses, logistic regression was used and all the fixed effects (including intercepts) were treated as random effects varying across subjects. For analyses that included more than one session, random effects were assumed to vary across sessions and subjects in a nested manner. Confidence intervals (CI) of the estimates were calculated using the ‘confint’ method of lme4 package with the ‘Wald’ parameter.

In the analyses of the stage 1 of non-probe sessions, we used a logistic regression analysis in which the independent predictor was whether the previous trial was rewarded (reward or no-reward), and the dependent variable was staying on the same stage 1 action. The *p*-value of this analysis was used in Fig 3a for colour-coding each bar, and the height of each bar represented the log odds ratio. The intercept term of this analysis is shown in S1 Fig. In the analyses of stage 1 of the probe sessions, the independent predictors were transition type of the previous trial (rare or common) and whether the previous trial was rewarded (reward or no-reward), whereas the dependent variable was staying on the same stage 1 action. The effects of interest were reward and the reward by transition-type interaction. In the analysis of stage 2 probe sessions, the independent variables were whether the stage 1 action was repeated (same stage 1), and whether the previous trial was rewarded. The dependent variable was staying on the same stage 2 action. The effect of interest was the interaction between the two independent variables. Note that only trials in which the stage 2 state was different from the stage 2 state of the previous trial were included in this analysis.

In all the analyses, only trials in which subjects made a correct discrimination on the previous trial (‘R’ in S2, and ‘L’ in S1) were included (%71 of trials in the whole training period). This was for two reasons. Firstly, it was not clear how subjects learn from actions taken during incorrect discriminations, which were never rewarded. Secondly, as depicted in Fig 3c, for the analysis of adaptive action representation, we focused on the trials in which the stage 2 states was different from that of the previous trial. When executing action sequences, we expected the subject to take the same stage 2 action in the next trial if (i) they were rewarded in the previous trial and (ii) they take the same stage 1 action, but not otherwise (as we focused on consecutive trials with different stage 2 states). However, assume that the subject makes an incorrect discrimination in the previous trial, e.g., it takes action ‘L’ at stage 1, moves to state S2 and takes action ‘L’ in that state, which is not rewarded since action ‘L’ in S2 is never rewarded. In the next trial, if the subject takes action ‘L’ again and ends up in state S1 (Fig 3c), there is a high chance that it will take ‘L’ again at stage 2, since ‘R’ is never rewarded in S1. Therefore, even if no reward was earned in the previous trial, there is a high chance that the subject will repeat the same stage 2 action in a different trial. This only happens in the condition that the subject made an incorrect discrimination in the previous trial, and in order to remove this interaction between the discrimination between actions at stage 2, and the analysis of action sequences, we only included the trials in which the subjects made correct discrimination in the previous trial. The analysis similar to the one presented in S4 Table without removing these trials is presented in S5 Table, which shows that the main statistical tests that we used to argue for adaptive state-space and action representations are statistically significant whether we include all of the trials or not.

### Computational modelling

Reinforcement-learning models considered for behavioural analysis were similar to the hierarchical RL models provided in [10] and the model-based/model-free family provided in [9]. In addition to these families, we also considered a family of hierarchical models (corresponding to the H family) in which only action sequences were available at stage 1 (i.e., single actions ‘L’ and ‘R’ were not available). In addition to the free-parameters mentioned in previous work, we added two new parameters here. The first free-parameter only applies to the hierarchical families (H, H-MB, H-MF, H-MB-MF), which represented the probability that the performance of action sequences is interrupted in the middle of the action sequence (i.e., subjects only perform the first component of an action sequence, and select a new action at stage 2). The other free-parameter coded the tendency of animals to take the discriminative action at stage 2, irrespective of the value of each action (tendency to take ‘R’ in S2 and ‘L’ in S1). This free-parameter allowed the model to learn that one of the actions in each of the stage 2 states was never rewarded. Details of the computational models along with their mathematical descriptions are presented in S1 Text. For the purpose of model comparison, we generated different instances of each family of models with different degrees of freedom (see S1 Text for details). Model-evidence, reported in Table 3 and Fig 4 was calculated similar to [35].

## Supporting information

S1 Text Computational modelling. (PDF)

S2 Text Supplementary experiments. (PDF)

S1 Table For the best model in each family, the table represents the negative log-model evidence (− log *P*(*D*|*M*)) for each model, the number of free parameters of each model (df), the free parameters of each model, and the family of each model. The table also represents a pseudo-r statistic (p–*r*^2^), which is a normalized measure of the variance accounted for in comparison to a model with random choices (averaged over subjects). ‘*’ indicated the model with the best model evidence.
(PDF)

S2 Table Value of the estimated parameters for each subject. (PDF)

S3 Table Total number of trials in the experiment reported in the main paper. (PDF)

S4 Table Results of the logistic regression analysis of stage 1 and stage 2 choices for the experiment reported in the main paper. For the stage 1 choices, the analysis is focused on staying on the same stage 1 action on the next trial, based on whether the previous trial was rewarded (reward), and whether the previous trial was common or rare (transition). ‘reward:transition’ is the interaction between reward, and transition type, and ‘intercept’ refers to the intercept term. For stage 2 choices, the analysis is focused on staying on the same stage 2 action, based on staying on the same stage 1 action (stay) and earning a reward in the previous trial (reward). ‘reward:stay’ is the interaction between ‘reward’, and ‘stay’. ‘probe 3’ are the results reported in the main paper.
(PDF)

S5 Table Results of the logistic regression analysis of stage 1 and stage 2 choices for the experiment reported in the main paper. For the stage 1 choices, the analysis is focused on staying on the same stage 1 action on the next trial, based on whether the previous trial was rewarded (reward), and whether the previous trial was common or rare (transition). ‘reward:transition’ is the interaction between reward, and transition type, and ‘intercept’ refers to the intercept term. ‘correct’ means that whether the correct stage 1 action was taken in the previous trial. ‘correct’ stage 1 action in refers to the stage 1 action which led the rewarded stage 2 state. For stage 2 choices, the analysis is focused on staying on the same stage 2 action, based on staying on the same stage 1 action (stay) and earning a reward in the previous trial (reward). ‘reward:stay’ is the interaction between ‘reward’, and ‘stay’. This table is different from S4 Table in two aspects: (i) the ‘correct’ predictor was added to the analysis following Akam et al’s [17] suggestion, (ii) unlike the analysis performed in S4 Table, the trials in which subjects did not make the correct discrimination were not excluded from the analysis. Note that the aborted trial, i.e., the trials in which animals entered the magazine between stage 1 and stage 2 actions were excluded. The reason for this second difference is, since rewards are deterministic (within each reversal), if we only include trials in which animals make correct discrimination, then ‘reward-transition’ predictor, and ‘correct’ predictor will be identical. As such, in this analysis that ‘correct’ is a predictor, we included all the trials.
(PDF)

S6 Table Results of the logistic regression analysis of stage 1, and stage 2 choices in supplementary experiment 1. For the stage 1 choices, the analysis is focused on staying on the same stage 1 action on the next trial based on whether the previous trial was rewarded (reward) and whether the previous trial was common or rare (transition). ‘reward:transition’ is the interaction between reward, and transition type. For stage 2 choices, the analysis focuses on staying on the same stage 2 action, based on staying on the same stage 1 action (stay), and earning a reward on the previous trial (reward). ‘reward:stay’ is the interaction between ‘reward’, and ‘stay’. ‘*p*’ refers to *p*-value. For the last probe session (s94) since the pattern of choices was not stationary but changing during the session, we presented separately the analysis for the first 16 earned outcomes during the sessions (s94:1; outcomes 1:16), second 16 outcomes earned during the session (s94:2; outcomes 17:32), and third 16 outcomes earned during the session (s94:3; outcomes 33:48).
(PDF)

S7 Table Total number of trials completed by the subjects in the supplementary experiment 1. (PDF)

S8 Table Results of the logistic regression analysis of stage 1 and stage 2 choices in supplementary experiments 2 and 3. For the stage 1 choices, the analysis is focused on staying on the same stage 1 action on the next trial, based on whether the previous trial was rewarded (reward), and whether the previous trial was common or rare (transition). ‘reward:transition’ is the interaction between reward, and transition type. For stage 2 choices, the analysis is focused on staying on the same stage 2 action, based on staying on the same stage 1 action (stay) and earning a reward in the previous trial (reward). ‘reward:stay’ is the interaction between ‘reward’, and ‘stay’.
(PDF)

S9 Table Total number of trials completed by the subjects in the supplementary experiments 2, 3. (PDF)

S1 Fig The graph shows the intercept term for the analysis shown in Fig 3a, which is the log odds ratio of staying on the same stage 1 action. The *p*-value of this analysis (for the intercept term) was used for colour-coding each bar. The graphs can be interpreted as the tendency of staying on the same stage 1 action, independent of whether reward was earn in the previous trial. The sessions marked with ‘strict sequence’ are similar to the sessions described in Fig 3a.
(PDF)

S2 Fig The graph shows the average session duration (across subjects). The animals could earn maximum of 60 outcomes and the session ended as soon as animals earned 60 outcomes. The session length was limited to an hour. The results are for the experiment reported in the main paper. Error-bars represent 1SEM.
(PDF)

S3 Fig Log odds ratio of the probability of staying on the same stage 1 action after getting rewarded on the previous trial (for the main experiment). Each panel shows the data for each subject. Log odds ratio = 0 implies an equal preference for both actions. The odds ratios are calculated using logistic regression and p-values are colour coded.
(PDF)

S4 Fig Results of discrimination training (for the experiment reported in the main paper) showing the percentage of correct responses averaged over subjects. Each point refers to a training session and error-bars are ±1 SEM.
(PDF)

S5 Fig Supplementary experiment 1. In this experiment several probe sessions were inserted in the middle of the training sessions in order illustrate the development of actions over the training period. (a) Log odds ratio of the probability of staying on the same stage 1 action after getting rewarded on the previous trial. Log odds ratio = 0 implies an equal preference for both actions. Sessions marked with ‘*’ are the probe sessions which included both rare and common transitions. (b-f) The probability of staying on the same stage 1 action in the probe sessions, averaged over subjects, as a function of whether the previous trial was rewarded (reward/no reward), and whether the transition in the previous trial was common or rare. The graphs illustrate a gradual shift from a simple state-space representation (panel b) to reward-guided actions (panels c,d), to goal-directed choices (panel e), and finally to a mixture of goal-directed and automatic actions (panel f). (g) Stage 2 actions in session s78. The graph shows the probability of staying on the same stage 2 action, averaged over subjects, as a function of whether the previous trial was rewarded (reward/no reward), and whether subjects stayed on the same stage 1 action (stay/switch). Similar to the analysis of the experiment reported in the main paper, only trials in which the stage 2 state is different from the previous trial are included in panel (g) in order to detect the performance of action sequences. Similarly, only trials in which subjects made a correct discrimination on the previous trial (‘R’ in S2, and ‘L’ in S1) were included in panels (a-g; see text). In all the probe sessions, the probability of rare transitions was 80%, except for the last session (s94) in which the probability of common and rare transitions was equal (i.e., 50%), in order to establish the effect of the transition probabilities on actions. (h) Results of initial discrimination training showing the percentage of correct responses averaged over subjects. Error-bars ±1 SEM.
(PDF)

S6 Fig Supplementary experiment 2. (a) The log odds ratio of staying on the same stage 1 action after earning a reward on the previous trial over the odds after earning no reward. Sessions denoted by ‘all rare’ included only rare transitions (similar to sessions marked with ‘#’ in Fig 3a). (b) The probability of staying on the same stage 1 action in the probe session (session s76) as a function of whether the previous trial was rewarded (reward/no reward) and whether the transition in the previous trial was common or rare. (c) The probability of staying on the same stage 2 action in the probe session (session s76), as a function of whether the previous trial was rewarded (reward/no reward) and whether subjects stayed on the same stage 1 action (stay/switch). Similar to the analysis in the main paper, only trials in which the stage 2 state was different from the previous trial are included in panels (c) in order to detect the performance of action sequences. Similarly, only trials in which subjects made a correct discrimination on the previous trial (‘R’ in S2, and ‘L’ in S1) were included in panels (a-c). In all the probe sessions the probability of rare transitions was 50%. (d) Results of discrimination training showing the percentage of correct responses. Error-bars ±1 SEM.
(PDF)

S7 Fig Supplementary experiment 3. (a) The log odds ratio of staying on the same stage 1 action after earning a reward on the previous trial over the odds after earning no reward. Sessions denoted by ‘all rare’ included only rare transitions (similar to sessions marked with ‘#’ in Fig 3a). ‘strict sequences’ indicates that trials with magazine responses after stage 1 actions were aborted. (b) The probability of staying on the same stage 1 action in the probe session (session s74) as a function of whether the previous trial was rewarded (reward/no reward), and whether the transition in the previous trial was common or rare. (c) The probability of staying on the same stage 2 action in the probe session (session s74), as a function of whether the previous trial was rewarded (reward/no reward), and whether subjects stayed on the same stage 1 action (stay/switch). Similar to the analysis presented in the main paper, only trials in which the stage 2 states were different from the previous trial are included in panels (c) in order to detect the performance of action sequences. Similar to the analysis in the main paper, only trials in which subjects made a correct discrimination on the previous trial (‘R’ in S2, and ‘L’ in S1) were included in panels (a-c). In the probe sessions, the probability of rare transitions was 50%. (d) Results of discrimination training showing the percentage of correct responses. Error-bars ±1 SEM.
(PDF)

S8 Fig Model simulations depicting the probability of staying on the same stage 2 action (left column) and the same stage 1 action (right column) using the best-fitted parameters for each subject in each model class. Note that similar to the other figures, in the left column only the trials in which state 2 state is different from the previous trial are included.
(PDF)
