# Supplementary material for: Learning the structure of the world: The adaptive nature of state-space and action representations in multi-stage decision-making
Source: PLoS Comput Biol. 2019 Sep 6;15(9):e1007334. doi: 10.1371/journal.pcbi.1007334 (PMC6750884; doi:10.1371/journal.pcbi.1007334)
Supplement: S1 Text — (PDF) [file pcbi.1007334.s001.pdf]

S1 Text – Computational modelling

We compared seven different families of the RL algorithms in order to evaluate which one provides a better explanation for data. These five families are: (1) a non-hierarchical model-based RL family (MB); (2) a hierarchical RL family (H); (3) a hierarchical model-based RL family (H-MB); (4) a model-free RL family (MF), (5) a hybrid model-based RL and model-free RL family (MB-MF), (6) a hierarchical model-free RL (H-MF), and (7) a hierarchical hybrid model-free and model-based RL (H-MB-MF). Each family had several instances, that we described them below.

We assumed that the environment has five states; the initial state denoted by S_0, stage 2 states denoted by S_1 and S_2, the reward state denoted by S_{Re} and no-reward state denoted by S_{NR}. For the case of non-hierarchical models, we assumed that actions L and R are available in states S_0, S_1 and S_2, and for the case of hierarchical models, we assumed actions L, R, LR, LL, RL, and RR are available in states S_0, and actions L and R, are available in states S_1 and S_2.

Model-based RL (MB)

Model-based RL (MB) is suggested to be the computational substrate for goal-directed decision-making. The model-based system works by learning the model of the environment, and then calculating the value of actions using the learned model. The model of the environment is composed of the transition function ($T(.)$), and the reward function ($R(.)$). We denote the transition function with $T(s'|a,s)$ which is the probability of reaching state s' after executing action a in state s. We assume that the transition function at the first stage is fixed,

$$T(\mathrm{S}_1|\mathrm{R},\mathrm{S}_0) = 0.8, T(\mathrm{S}_2|\mathrm{L},\mathrm{S}_0) = 0.8, \tag{1}$$

and it will not change during learning. For the other states, after executing action a in state s and reaching state s', the transition function updates as follows:

$$\forall s'' \in \{\mathrm{S}_{\mathrm{Re}}, \mathrm{S}_{\mathrm{NR}}\} : \tag{2}$$

$$T(s''|s,a) = \begin{cases} (1-\eta)T(s''|s,a) + \eta & : s' = s'' \\ (1-\eta)T(s''|s,a) & : s' \neq s'' \end{cases}, \tag{3}$$

where $\eta (0 < \eta < 1)$ is the update rate of the state-action-state transitions. For the reward functions, we assumed that the reward at state S_{Re} is one, and zero in all other states,

$$R(s) = \begin{cases} 1 : & s = \mathrm{S}_{\mathrm{Re}} \\ 0 : & \text{otherwise} \end{cases}. \tag{4}$$

Based on the above reward and transition functions, the goal-directed (model-based) value of taking action a in state s is as follows:

$$\forall s \in \{\mathrm{S}_0, \mathrm{S}_1, \mathrm{S}_2\} : Q^{\mathrm{MB}}(s,a) = \sum_{s'} T(s'|s,a) V^{\mathrm{MB}}(s'), \tag{5}$$

where:

$$V^{\mathrm{MB}}(s) = \begin{cases} \max_a Q^{\mathrm{MB}}(s,a) & : s \in \{\mathrm{S}_0, \mathrm{S}_1, \mathrm{S}_2\} \\ R(s) & : s \in \{\mathrm{S}_{\mathrm{Re}}, \mathrm{S}_{\mathrm{NR}}\} \end{cases} . \tag{6}$$

Finally, the agent uses the calculated values to choose actions. The probability of selecting action a in state s, denoted by $\pi(s,a)$, will be determined according to the soft-max rule:

$$\pi(s,a) = \frac{e^{\beta(s)Q^{\mathrm{MB}}(s,a)+\kappa(s,a)+d(s,a)}}{\sum_{a'} e^{\beta(s)Q^{\mathrm{MB}}(s,a')+\kappa(s,a')+d(s,a')}}. \tag{7}$$

The above equation reflects the fact that actions with higher values are more likely to be selected. The $\beta(s)$ parameter controls the rate of exploration; the parameter $\kappa(s,a)$ is the action preservation parameter and captures the general tendency of taking the same action as the previous trial [23, 30]. Finally, the term $d(s,a)$ represents the tendency of the subjects to take the discriminative actions at the stage 2 states (taking action R in S_2 and action L in S_1). A positive value for this parameter entails that a subject has a tendency to take the discriminative action at stage 2 states. Note that the effect of this parameter is on top of the effect of values of the actions at the stage 2 states.

For the exploration parameter, we assume that $\beta(s) = \beta_1$ if $s = \mathrm{S}_0$ and $\beta(s) = \beta_2$ if $s \in \{\mathrm{S}_1, \mathrm{S}_2\}$. For the perseveration parameter we assumed that if $s = \mathrm{S}_0$ and a being the action taken in the previous trial in the S_0 state, then $\kappa(s,a) = k$, otherwise it will be zero. Finally, for the discrimination parameter, we assume

$$d(s,a) = \begin{cases} \phi & : (s,a) \in \{(\mathrm{S}_1, \mathrm{L}), (\mathrm{S}_2, \mathrm{R})\} \\ 0 & : \text{otherwise} \end{cases} . \tag{8}$$

In the most general form, all the parameters $(\beta_1, \beta_2, \eta, k, \phi)$ were treated as free parameters. We also generated eight variants by (1) setting $\beta_1 = \beta_2$ (i.e., rate of exploration at stage 1 and stage 2 states are the same), (2) setting $k = 0$ (there is no tendency to perseverate on the previously taken actions), and (3) setting $\phi = 0$ (there is no tendency to take the discriminative action at stage 2).

Hierarchical model-based RL (H-MB)

Implementation of the hierarchical structure is similar to hierarchical RL, with action sequences (LL, LR, etc) as *options* (equivalent to action sequences in this setting). We assumed actions L, R, LL, LR, RL, and RR are available in states S_0, and actions L and R, are available in states S_1 and S_2. After reaching a terminal state (S_{Re} or S_{NR}), transition functions of both the action sequence, and the single action that led to that state update according to equation 3. In the case of single actions, the transition function will be updated by the $\eta = \eta_1$ update rate, and in the case of action sequences, the transition function will be updated by the $\eta = \eta_2$ update rate. Based on the learned transition function, value of actions in each state is calculated by the goal-directed system using equation 5. Using the state-action values ($V^{\mathrm{MB}}(s,a)$), the probability of selecting each action under goal-directed control will be as follows:

$$\pi(s,a) = \frac{e^{\beta(s,a)Q^{\mathrm{MB}}(s,a)+\kappa(s,a)+d(s,a)}}{\sum_{a'} e^{\beta(s,a)Q^{\mathrm{MB}}(s,a')+\kappa(s,a')+d(s,a')}}, \tag{9}$$

where $\beta(s,a)$ is the rate of exploration. The rate of exploration for stage 2 actions ($s \in \{S_1, S_2\}$) is $\beta(s,a) = \beta_2$. For stage 1 actions ($s = S_0$), if a is a single action, we assume $\beta(s,a) = \beta_1$, and if a is an action sequence $\beta(s,a) = \beta_3$:

$$\beta(s,a) = \begin{cases} \beta_1 & : s \in \{\mathrm{S}_0\} \text{ and } a \in \{\mathrm{L}, \mathrm{R}\} \\ \beta_2 & : s \in \{\mathrm{S}_1, \mathrm{S}_2\} \\ \beta_3 & : s \in \{\mathrm{S}_0\} \text{ and } a \in \{\mathrm{LL}, \mathrm{RR}, \mathrm{RL}, \mathrm{LR}\} \end{cases} . \tag{10}$$

Note that we set $\beta_3 = \beta_1$.

As before, $\kappa(s,a)$ captures action perseveration. We assumed that $\kappa(s,a) = k_1$ if action a is a single action, and $\kappa(s,a) = k_2$ if action a is an action sequence:

$$\kappa(s,a) = \begin{cases} k_1 & : s \in \{\mathrm{S}_0\} \text{ and } a \in \{\mathrm{L}, \mathrm{R}\} \\ k_2 & : s \in \{\mathrm{S}_0\} \text{ and } a \in \{\mathrm{LL}, \mathrm{RR}, \mathrm{RL}, \mathrm{LR}\} \\ 0 & : \text{otherwise} \end{cases} . \tag{11}$$

Parameter $d(s,a)$ is similar to the one defined in the previous section.

For calculating the probability of selecting each action, equation 5 was used in the case of stage 1 actions, as these actions are chosen using using model-based valuation. In the case of stage 2 actions, however, the probability of selecting actions depends on whether the action to be executed is part an action sequence selected earlier, or is it a single action selected at stage 2 based on the model-based valuations at this stage, i.e., if the action selected at stage 1 is a single action then the action at stage 2 will be selected using equation 5, however, if the action selected at stage 1 is an action sequence, then the second component of the selected action sequence will be executed in stage 2. Based on this, we calculated the probabilities of selecting actions at stage 2 as follows. Assume we know action L has been executed in state S_0 by the subject; then, the probability of this action being due to performing the LR action sequence is:

$$p(\mathrm{LR}|\mathrm{S}_0, \mathrm{L}) = \frac{\pi(\mathrm{LR}|\mathrm{S}_0)}{\pi(\mathrm{L}|\mathrm{S}_0) + \pi(\mathrm{LR}|S_0) + \pi(\mathrm{LL}|\mathrm{S}_0)}. \tag{12}$$

Similarly, the probability of observing L due to selecting the single action L at stage 1 is:

$$p(\mathrm{L}|\mathrm{S}_0, \mathrm{L}) = \frac{\pi(\mathrm{L}|\mathrm{S}_0)}{\pi(\mathrm{L}|\mathrm{S}_0) + \pi(\mathrm{LR}|\mathrm{S}_0) + \pi(\mathrm{LL}|\mathrm{S}_0)}. \tag{13}$$

Based on this, the probability that the model assigns to action a in state $s \in \{\mathrm{S}_1, \mathrm{S}_2\}$, given that action a' is being observed in S_0 is:

$$p(a|s) = p(a'|S_0, a')\pi(a|s) + p(a'a|S_0, a'), \tag{14}$$

where $p(aa'|\mathrm{S}_0, a')$ and $p(a'|\mathrm{S}_0, a')$ are calculated using equations 12 and 13 respectively.

Next, we assumed that even under the conditions in which an action sequence is being executed, there is a chance that the performance of the action sequence will be interrupted at stage 2, that is, a subject selects an action sequence at stage 1, but stops executing the action sequence at stage 2, and selects a new action using model-based evaluations (equation 5). This variant is inspired by similar approaches in the hierarchical RL literature (see Hengst [21] for a review).

Let's assume that the probability of interrupting an action sequence is I, then equation 14 will become as follows:

$$p(a|s) = \pi(a|s)\big(p(a'|\mathrm{S}_0, a')(1-I) + I\big) + (1-I)p(a'a|\mathrm{S}_0, a'). \tag{15}$$

It can be verified that in the case of $I = 0$, i.e., action sequences never become interrupted, the above equation will degenerate to equation 14. In the case of $I = 1$, i.e., all the action sequences are interrupted and they have no effect on stage 2 choices, and we have:

$$p(a|s) = \pi(a|s), \tag{16}$$

which indicates that the probability of taking each action at stage 2 is guided only by the rewards earned on that stage 2, and not by the action sequences in the first stage.

In the most general form, all the free parameters are included in the model: β_1 ($\beta_3 = \beta_1$), β_2, η_1, η_2, k_1, k_2, ϕ, I. We generated 64 simpler models by setting (1) $\beta_1 = \beta_2$ (exploration rates at stage 1 and stage 2 choices are the same), (2) $\eta_1 = \eta_2$ (learning rates for action sequences and single actions are the same), (3) $k_1 = 0$ (no perseveration for single actions), (4) $k_2 = 0$ (no perseveration for action sequences), (5) $\phi = 0$ (no tendency to take discriminative actions), (6) $I = 0$ (action sequences are never interrupted).

Hierarchical (H)

This family is similar to H-MB family, except that only action sequences (LL, LR, RL, RR) can be selected at stage 1 (S_0). In the most general form the free-parameters included β_1 (exploration parameter at stage 1), β_2 (exploration parameter at stage 2), η_1 (learning rate for action/action sequences), k_2 (preservation on action sequences), ϕ (discrimination parameter), I (sequence interruption parameter). We then generated 16 different variants by setting $\beta_1 = \beta_2$, $k_2 = 0$, $\phi = 0$, $I = 0$.

Model-free RL (MF)

We used Q-learning [44] for model-free learning. After taking action a in state s, and reaching state s', the model-free values update as follows:

$$Q^{\mathrm{MF}}(s,a) \leftarrow Q^{\mathrm{MF}}(s,a) + \alpha(s)(V^{\mathrm{MF}}(s') - Q^{\mathrm{MF}}(s,a)), \tag{17}$$

where $\alpha(s)(0 < \alpha(s) < 1)$ is the learning rate, which can be different in stage 1 and stage 2 states,

$$\alpha(s) = \begin{cases} \alpha_1 & : s = \mathrm{S}_0 \\ \alpha_2 & : s \in \{\mathrm{S}_1, \mathrm{S}_2\} \end{cases}. \tag{18}$$

In equation 17, $V^{\mathrm{MF}}(s)$ is the value of the best action in state s:

$$V^{\mathrm{MF}}(s) = \begin{cases} \max_a Q^{\mathrm{MF}}(s,a) & : s \in \{\mathrm{S}_0, \mathrm{S}_1, \mathrm{S}_2\} \\ R(s) & : s \in \{\mathrm{S}_{\mathrm{Re}}, \mathrm{S}_{\mathrm{NR}}\} \end{cases}. \tag{19}$$

In addition to the update in equation 17 after taking actions at stage 2, the value of the action that was taken at

stage 1 will also get updated according to the outcome. Assume a is the action that was taken in S_0, a' is the action that was subsequently taken in s (second stage states, i.e., $s \in \{\mathrm{S}_0, \mathrm{S}_1\}$), and s' is the state that was visited after executing a' ($s' \in \{\mathrm{S}_{\mathrm{Re,NR}}\}$). Then, action values update as follows:

$$Q^{\mathrm{MF}}(\mathrm{S}_0, a) \leftarrow Q^{\mathrm{MF}}(\mathrm{S}_0, a) + \alpha_1 \lambda (V^{\mathrm{MF}}(s') - Q^{\mathrm{MF}}(s, a')), \tag{20}$$

where $\lambda (0 < \lambda < 1)$ is the reinforcement eligibility parameter, and it determines the extent to which the first stage action values are affected by receiving the outcome after executing the second stage actions. The action selection method, and variants of this form of learning are described in the next section.

Model-free, model-based hybrid RL (MF-MB)

This model is a combination of model-free RL, and model-based RL, in which final action values are computed by combining the values provided by model-free and model-based processes,

$$Q(s, a) = wQ^{\mathrm{MB}}(s, a) + (1 - w)Q^{\mathrm{MF}}(s, a), \tag{21}$$

were $w(0 < w < 1)$ determines the relative contribution of model-free and model-based values into the final values.

The probability of selecting action a in state s will be determined according to the soft-max rule:

$$\pi(s, a) = \frac{e^{\beta(s)Q(s,a) + \kappa(s,a) + d(s,a)}}{\sum_{a'} e^{\beta(s)Q(s,a') + \kappa(s,a') + d(s,a')}}, \tag{22}$$

where parameters are same as the ones we described in the Model-based RL (MB) section.

In the most general form, all the free parameters are included in the model: β_1, β_2, η, α_1, λ, w, k, ϕ (we assumed that $\alpha_2 = \eta$). We generated 32 simpler models by setting (1) $\lambda = 0$, (2) $\alpha_1 = \alpha_2$ (learning rate of model-free system is the same at stage 1 and stage 2 states), (3) $\beta_1 = \beta_2$ (rate of exploration is the same at stage 1 and stage 2 states), (4) $k = 0$ (there is no tendency to perseverate on the previously taken action), and (5) $\phi = 0$ (there is no tendency to take the discriminative action at stage 2).

By setting $w = 0$ the above hybrid model degenerated to a model-free process described in the previous section, and therefore, we generated 32 variants of model-free RL (similar to the hybrid model), by setting $w = 0$.

Hierarchical model-free RL (H-MF)

This model is similar to hierarchical model-based RL except that the Q-values are calculated using model-free RL instead of model-based RL. The method for calculating model-free values are described in the previous section. Note that in this model at state S_0 both single actions and action sequences are available. The rate of update of $Q-$values of action sequences is α_2. In the most general form, the free-parameters include β_1, β_2, α_1, α_2, λ, k, ϕ, k_2 (perseveration for actions sequences), I.

We generated 128 simpler models by setting (1) $\lambda = 0$, (2) $\alpha_1 = \alpha_2$ (learning rate of model-free system is the same at stage 1 and stage 2 states), (3) $\beta_1 = \beta_2$ (rate of exploration is the same at stage 1 and stage 2 states), (4) $k = 0$ (there is no tendency to perseverate on the previously taken action), and (5) $\phi = 0$ (there is no tendency to take the

discriminative action at stage 2), (6) $k_2 = 0$ (no perseveration for actions sequences), and (7) $I = 0$ (sequences are never interrupted).

Hierarchical model-based/model-free RL (H-MB-MF)

This model is similar similar to model-free, model-based hybrid RL, but both actions and action sequences are available to the agent in state S_0. In the most general form, the free-parameters include β_1, β_2, η_1, η_2, α_1, λ, k, ϕ (we assumed that $\alpha_2 = \eta_1$), k_2 (perseveration for actions sequences), w, and I

We generated 256 simpler models by setting (1) $\lambda = 0$, (2) $\alpha_1 = \alpha_2$ (learning rate of model-free system is the same at stage 1 and stage 2 states), (3) $\beta_1 = \beta_2$ (rate of exploration is the same at stage 1 and stage 2 states), (4) $k = 0$ (there is no tendency to perseverate on the previously taken action), and (5) $\phi = 0$ (there is no tendency to take the discriminative action at stage 2), (6) $k_2 = 0$ (no perseveration for actions sequences), (7) $\eta_1 = \eta_2$, and (8) $I = 0$ (sequences are never interrupted).

Model comparison

We took a hierarchical Bayesian approach to compare different models. This approach provides a framework to compare models based on their complexity and their fit to data. Bayesian model comparison is based on the model evidence quantity, which is the probability of the data given a model. The approach that we took to calculate this quantity is similar to the approach taken in Piray et al. [37].

For each model, there are two sets of free parameters: group-level parameters denoted by Θ (we call these parameters hyper-parameters), and subject-level parameters, denoted with θ_i for subject i. The hyper-parameters define the prior distribution over subject-level parameters. The aim is to calculate the probability of data (denoted by D) given model M:

$$P(D|M) = \int P(D|M, \Theta)P(\Theta)d\Theta, \tag{23}$$

Since the above integral is intractable, we approximate it using Bayesian Information Criterion (BIC) [40]:

$$\log P(D|M) \approx \sum_i \log P(D_i|M, \Theta^{\mathrm{ML}}) - \frac{1}{2}|\Theta| \log |D|, \tag{24}$$

where Θ^{ML} is the maximum-likelihood estimate of Θ, and D_i is the data of subject i. $|\Theta|$ is the number of hyper-parameters, and $|D|$ is the sum of number of choices made by all the subjects. In the above formula, the term inside the sum is:

$$P(D_i|M, \Theta^{\mathrm{ML}}) = \int P(D_i|M, \theta_i)P(\theta_i|\Theta^{\mathrm{ML}})d\theta_i, \tag{25}$$

which is again intractable to compute, and we use Laplace method [32] to approximate it:

$$\log P(D_i|M, \Theta^{\mathrm{ML}}) \approx \log P(D_i|M, \theta_i^{\mathrm{MAP}}) + \tag{26}$$

$$\log P(\theta_i^{\mathrm{MAP}}|\Theta^{\mathrm{ML}}) + \frac{1}{2}|\theta_i| \log 2\pi - \frac{1}{2}\log |H_i|, \tag{27}$$

where θ_i^{MAP} is the maximum a posterior (MAP) estimate of θ_i. $|\theta_i|$ is the number of free parameters for model M,

and $|H_i|$ is determinant of the Hessian matrix at θ_i^{MAP}.

Thus in summary, we calculated the model evidence for each subject using equation 27, and then we summed over all the model evidence for all the subjects to calculate equation 24, which is the model evidence over the whole group.

The only remaining question is how to calculate Θ^{ML}, which is:

$$\Theta^{\text{ML}} = \arg\max_{\Theta} \sum_i \log \int P(D_i|M, \theta_i) P(\theta_i|\Theta) d\theta_i. \tag{28}$$

Similar to Huys et al. [22], we solved the above optimization problem using the expectation-maximization (EM) procedure [13]. This procedure starts with an initial value for the hyper-parameters Θ, using which the posterior distribution of each individual's parameter will be estimated using the Laplace approximation. These individual posterior distributions then shape a new value for the hyper-parameters (Θ), which will be used again to get new posterior distribution for each individual. This process continues until the hyper-parameters do not change anymore across iterations. Please refer to Huys et al. [22] for the details of the method.

The prior over all the individual level parameters (θ_i) where assumed to be a Gaussian distribution, and the mean and variance of the Gaussian were included in the hyper-parameters (thus the number of hyper-parameters for each model were twice as the number of free parameters of the model). Parameters that had a limited range (e.g., learning rates), were transformed to satisfy the constrains.

We used the NLOPT software package [26] for nonlinear optimization using 'BOBYQA' algorithm. Finally, we used 'DerApproximator' package [28] in order to estimate the Hessian at the MAP point.

Model comparison results

In total we tested 536 models (H-MB-MF:n=256, H:n=16, MB:n=8, MF:n=32, MB-MF:n=32, H-MF:n=128, MB-MB:n=64). Table S1 shows the different properties of the best model in each family. Out of 536 models that we tested three models were not identifiable (the estimated Hessian matrix was not a positive-definite matrix), and therefore it was excluded from the analysis. Table S2 represents estimated parameters for each individual in the best model (indicated by * in Table S1). The term '$-\log P(D_i|M, \Theta^{\text{ML}})$' represents the negative log-model evidence for each subject, obtained from equation 27.
