# Supplementary material for: Learning the structure of the world: The adaptive nature of state-space and action representations in multi-stage decision-making
Source: PLoS Comput Biol. 2019 Sep 6;15(9):e1007334. doi: 10.1371/journal.pcbi.1007334 (PMC6750884; doi:10.1371/journal.pcbi.1007334)
Supplement: S2 Text — (PDF) [file pcbi.1007334.s002.pdf]

S2 Text – Supplementary experiments

The aim of this section is to present the results of three supplementary experiments (1-3) conducted using different parameters to the ones used for the experiment reported in the main paper. Each experiment shows that similar to Figure 3a there was a shift in the pattern of choices from simple representations to more complex representations that reflect the correct structure of the environment. In addition to this, we also report the results of the probe sessions conducted in these experiments. Those results reveal how the pattern of choices is affected by the variations in the parameters.

The parameters used in these experiments are different from the parameters used in the main paper in two respects. Firstly, the inter-trial interval (ITI) in these supplementary experiments was non-zero, whereas the ITI in the experiment reported in the main paper was zero. Secondly, in the supplementary experiments auditory cues (clicker and tone) were used to signal stage 2 states, whereas in the experiments reported in the main paper visual cues were used to signal stage 2 states. See Behavioural procedures below for the details of the parameters used in each experiment.

In the supplementary experiments, because the ITI was non-zero, the stage 1 state (S0) was explicitly signalled to the animals at the end of the ITI by the illumination of the house light. We assumed that the first response made after the ITI was the state 1 action and the response after that was the stage 2 action (and this is what stage 1 actions and stage 2 actions refer to in analysis presented below). The data, however, indicated that animals did not wait for the presentation of the stage 1 state to make their first response and made a significant number of responses during the ITI. These were unrewarded regardless of which action/action sequence was made by the animals. Clearly, this could interfere with the formation and/or expression of action sequences and, therefore, in the experiment reported in the main paper an ITI of zero was used to remove such interference. Furthermore, we used visual cues in the experiment reported in the main paper instead of the auditory cues used in the supplementary experiments. This was because the visual cues (used in the experiment reported in the main paper) are presumably less salient and so harder to discriminate than the auditory cues used in the supplementary experiments; it took twice the number of training sessions for the animals to learn the discrimination between the visual cues compared to the auditory cues (comparing Figure S4 with Figure S5h). Because of this reduction in slaience, when visual cues were being used less interference was introduced during the transition between states and so there was a higher tendency for the rats to perform the stage 1 and stage 2 actions in a sequence (uninterrupted) independent of the stage 2 states, increasing the opportunity to detect action sequences. For this reason, in the experiment reported in the main paper we used visual cues.

Subjects and apparatus

Details are similar to the experiment reported in the main paper (see section Subjects and apparatus).

Behavioural procedures

The general structure of the experiments was similar to the experiment reported in the main paper and it is depicted in Figure 1. Below we explain each step and highlight the similarities and differences between the experiments.

Magazine training, lever-press training, and discrimination training phases (phases 1-3) were similar to the experiment reported in the main paper (see section Material and Methods), except that in the supplementary experiments, the stimuli were the tone and clicker, but in the experiment reported in the main paper, the stimuli were a constant and a blinking house light.

Next, the rats received training on the two-stage task in which animals first made a binary choice at stage 1 (signalled by the illumination of the house light), after which they transitioned to the stage 2 state, in which again they made another binary choice that could lead to either reward delivery or no-reward. In supplementary experiments 1 and 2 there was a 20-s inter-trial interval (ITI) before the next trial started, and there was a 5-second ITI in supplementary experiment 3. Stage 2 states were signalled by the stimuli trained in the previous phase of the experiment. The stage 2 states were presented immediately after stage 1 actions were taken, and the reward/no-reward was presented immediately after the stage 2 action was taken.

In each trial, only one of the stage 2 states led to reward, whereas the other state did not lead to reward irrespective of the choice of actions. The stage 2 states that earned a reward frequently switched between states during the course of a session (Figure 2a). In supplementary experiment 1, this switch occurred after every four outcomes with a maximum 40 outcomes in a session, which later in the training increased to every eight outcomes as shown in Figure S5a (with a maximum 48 outcomes in a session and maximum duration of a session was limited to an hour). In supplementary experiment 2, the switch occurred whenever a randomly selected number of outcomes were received since the last switch. This random number was uniformly drawn from within a range from 8 to 16 outcomes (maximum 48 outcomes in a session and maximum duration of a session was limited to an hour). In supplementary experiment 3, the switch occurred every fourth outcome received (maximum 50 outcomes in a session and maximum duration of a session was limited to an hour). Furthermore, because the ITI was long in supplementary experiments 1 and 2, animals received a pre-training phase on the two-stage task in which the reward in the stage 2 states was fixed during a session, and was changed across sessions. Subjects received ten training sessions in this manner. Similarly, in supplementary experiment 2, subjects received two pre-training sessions in which they could earn a reward in both stage 2 states.

Animals were trained on the two-stage task for 69 sessions in supplementary experiment 1, 57 sessions in supplementary experiment 2, 60 sessions in supplementary experiment 3. In the middle of, or at the end of these training sessions, animals were given probe test sessions in which stage 1 actions led to stage 2 states in a probabilistic manner. One stage 1 action led to its specific stage 2 state 80% of the time, whereas the other stage 1 action led to the other stage 2 state (Figure 2c). For the last probe session in Experiments 1 and 3, the probability that stage 1 actions led to the corresponding stage 2 states was 50%. The exact positions of probe sessions for supplementary experiments 1 to 3 are depicted in Figures S5b, S6b, S7b respectively marked with an asterisk.

Results

Results for supplementary experiments 1-3 are shown in Figures S5, S6, S7 respectively. Multiple probe sessions were conducted in supplementary experiment 1 (marked with '*' in Figure S5b) and the statistical analysis of probe sessions are shown in Table S6. Supplementary experiments 2,3 contained a single probe session (marked with * in Figure S6b, and Figure S7b), and the statistical analysis of these probe sessions are shown in Table S8. Note that the

differences in the results of the probe sessions of the supplementary experiments compared to the those reported in the main paper is most likely due to the interference produced by unrewarded actions taken during the ITI (since the ITI was non-zero in the supplementary experiments), and also due to the use of auditory cues in the supplementary experiments (see above).

Similar to the experiment reported in main paper, at the beginning of training, as Figure S5a, S6a, S7a show, the rats not only failed to show a tendency to take the same action after earning a reward in the previous trial, they also showed a tendency to switch to the other action. This effect was statistically significant in the first five sessions of supplementary experiment 2 (sessions s20 to s24; $\beta = -0.253$ (CI: -0.371, -0.135), SE=0.060, p$<10^{-4}$) and supplementary experiment 3 (sessions s15 to s19; $\beta = -0.222$ (CI: -0.357, -0.086), SE=0.068, p=0.001); but, for supplementary experiment 1, although the effects were in the same direction, it was not statistically significant (sessions s26 to s30; $\beta = -0.063$, SE=0.045, p=0.161), likely due to the pre-training the animals received in that experiment.
