# Supplementary material for: Learning the structure of the world: The adaptive nature of state-space and action representations in multi-stage decision-making
Source: PLoS Comput Biol. 2019 Sep 6;15(9):e1007334. doi: 10.1371/journal.pcbi.1007334 (PMC6750884; doi:10.1371/journal.pcbi.1007334)
Supplement: S1 Table — The table also represents a pseudo-r statistic (p–r2), which is a normalized measure of the variance accounted for in comparison to a model with random choices (averaged over subjects). ‘*’ indicated the model with the best model evidence. (PDF) [file pcbi.1007334.s003.pdf]

Table S1. For the best model in each family, the table represents the negative log-model evidence ($-\log P(D|M)$) for each model, the number of free parameters of each model (df), the free parameters of each model, and the family of each model. The table also represents a pseudo-r statistic (p–r^2), which is a normalized measure of the variance accounted for in comparison to a model with random choices (averaged over subjects). '*' indicated the model with the best model evidence.

model family	free-parameters	p-r^2	df	$-\log p(D\|M)$
H-MB*	β_1, η_1, k_2, ϕ ($I = 0$)	0.207	4	1172.357
H	β_1, η_1, k_2, ϕ, I	0.207	5	1185.274
MF	β_1, β_2, α_1, λ	0.175	4	1219.87
MB	β_1, β_2, η, k	0.175	4	1222.57
MB-MF	β_1, β_2, α_1, λ, w	0.180	5	1223.515
H-MF	β_1, α_1, k_2, ϕ	0.204	4	1179.717
H-MB-MF	β_1, α_1, w, ϕ, k_2	0.211	5	1178.339
