# Supplementary material for: Learning the structure of the world: The adaptive nature of state-space and action representations in multi-stage decision-making
Source: PLoS Comput Biol. 2019 Sep 6;15(9):e1007334. doi: 10.1371/journal.pcbi.1007334 (PMC6750884; doi:10.1371/journal.pcbi.1007334)
Supplement: S2 Table — (PDF) [file pcbi.1007334.s004.pdf]

Table S2. Value of the estimated parameters for each subject.

subject	df	p-r^2	no. choices	$-\log p(D\|M, \Theta^{\mathrm{ML}})$	β_1	η_1	ϕ	k_2
0	4	0.255	244	128.08	1.9594	0.832	1.265	0.662
1	4	0.303	214	105.000	2.082	0.717	2.444	0.808
2	4	0.1833	264	150.594	1.632	0.755	2.130	0.670
3	4	0.317	206	98.936	1.902	0.748	2.607	0.650
4	4	0.064	286	188.287	0.972	0.756	1.916	0.460
5	4	0.197	272	152.832	1.597	0.667	2.310	0.653
6	4	0.190	296	168.495	1.376	0.821	2.300	0.931
7	4	0.151	252	149.657	1.258	0.772	2.219	0.616
