# Supplementary material for: Learning the structure of the world: The adaptive nature of state-space and action representations in multi-stage decision-making
Source: PLoS Comput Biol. 2019 Sep 6;15(9):e1007334. doi: 10.1371/journal.pcbi.1007334 (PMC6750884; doi:10.1371/journal.pcbi.1007334)
Supplement: S3 Table — (PDF) [file pcbi.1007334.s005.pdf]

Table S3. Total number of trials in the experiment reported in the main paper.

session	mean (SD)
probe 1	142.75(20.81)
probe 2	157.25(44.85)
probe 3	136.5(18.45)
