# Supplementary material for: Learning the structure of the world: The adaptive nature of state-space and action representations in multi-stage decision-making
Source: PLoS Comput Biol. 2019 Sep 6;15(9):e1007334. doi: 10.1371/journal.pcbi.1007334 (PMC6750884; doi:10.1371/journal.pcbi.1007334)
Supplement: S4 Table — For the stage 1 choices, the analysis is focused on staying on the same stage 1 action on the next trial, based on whether the previous trial was rewarded (reward), and whether the previous trial was common or rare (transition). ‘reward:transition’ is the interaction between reward, and transition type, and ‘intercept’ refers to the intercept term. For stage 2 choices, the analysis is focused on staying on the same stage 2 action, based on staying on the same stage 1 action (stay) and earning a reward in the previous trial (reward). ‘reward:stay’ is the interaction between ‘reward’, and ‘stay’. ‘probe 3’ are the results reported in the main paper. (PDF) [file pcbi.1007334.s006.pdf]

**Table S4.** Results of the logistic regression analysis of stage 1 and stage 2 choices for the experiment reported in the main paper. For the stage 1 choices, the analysis is focused on staying on the same stage 1 action on the next trial, based on whether the previous trial was rewarded (reward), and whether the previous trial was common or rare (transition). 'reward:transition' is the interaction between reward, and transition type, and 'intercept' refers to the intercept term. For stage 2 choices, the analysis is focused on staying on the same stage 2 action, based on staying on the same stage 1 action (stay) and earning a reward in the previous trial (reward). 'reward:stay' is the interaction between 'reward', and 'stay'. 'probe 3' are the results reported in the main paper.

| Stage 1 actions | | | | |
|---|---|---|---|---|
| | | probe 1 | probe 2 | probe 3 |
| intercept | $p$-value | 0.618 | 0.054 | 0.373 |
| | $\beta$ (SE) | -0.065 (0.131) | 0.299 (0.155) | -0.166 (0.186) |
| reward | $p$-value | 0.002 | <1e-4 | 0.003 |
| | $\beta$ (SE) | 0.52 (0.174) | 0.504 (0.128) | 0.612 (0.208) |
| transition | $p$-value | 0.832 | 0.155 | 0.164 |
| | $\beta$ (SE) | -0.057 (0.273) | -0.406 (0.285) | 0.371 (0.267) |
| reward:transition | $p$-value | 0.005 | 0.02 | <1e-5 |
| | $\beta$ (SE) | 0.842 (0.3) | 0.399 (0.171) | 0.923 (0.204) |
| Stage 2 actions | | | | |
| | | probe 1 | probe 2 | probe 3 |
| intercept | $p$-value | 0.221 | 0.576 | 0.001 |
| | $\beta$ (SE) | -0.266 (0.217) | -0.168 (0.301) | -0.95 (0.295) |
| reward | $p$-value | <1e-4 | <1e-5 | <0.001 |
| | $\beta$ (SE) | 0.631 (0.149) | 1.244 (0.265) | 0.849 (0.235) |
| stay | $p$-value | 0.111 | 0.255 | 0.019 |
| | $\beta$ (SE) | 0.254 (0.159) | 0.234 (0.205) | 0.608 (0.259) |
| reward:stay | $p$-value | 0.222 | 0.153 | 0.027 |
| | $\beta$ (SE) | 0.182 (0.149) | 0.29 (0.203) | 0.494 (0.224) |
