# Supplementary material for: Learning the structure of the world: The adaptive nature of state-space and action representations in multi-stage decision-making
Source: PLoS Comput Biol. 2019 Sep 6;15(9):e1007334. doi: 10.1371/journal.pcbi.1007334 (PMC6750884; doi:10.1371/journal.pcbi.1007334)
Supplement: S5 Table — For the stage 1 choices, the analysis is focused on staying on the same stage 1 action on the next trial, based on whether the previous trial was rewarded (reward), and whether the previous trial was common or rare (transition). ‘reward:transition’ is the interaction between reward, and transition type, and ‘intercept’ refers to the intercept term. ‘correct’ means that whether the correct stage 1 action was taken in the previous trial. ‘correct’ stage 1 action in refers to the stage 1 action which led the rewarded stage 2 state. For stage 2 choices, the analysis is focused on staying on the same stage 2 action, based on staying on the same stage 1 action (stay) and earning a reward in the previous trial (reward). ‘reward:stay’ is the interaction between ‘reward’, and ‘stay’. This table is different from S4 Table in two aspects: (i) the ‘correct’ predictor was added to the analysis following Akam et al’s [17] suggestion, (ii) unlike the analysis performed in S4 Table, the trials in which subjects did not make the correct discrimination were not excluded from the analysis. Note that the aborted trial, i.e., the trials in which animals entered the magazine between stage 1 and stage 2 actions were excluded. The reason for this second difference is, since rewards are deterministic (within each reversal), if we only include trials in which animals make correct discrimination, then ‘reward-transition’ predictor, and ‘correct’ predictor will be identical. As such, in this analysis that ‘correct’ is a predictor, we included all the trials. (PDF) [file pcbi.1007334.s007.pdf]

Table S5. Results of the logistic regression analysis of stage 1 and stage 2 choices for the experiment reported in the main paper. For the stage 1 choices, the analysis is focused on staying on the same stage 1 action on the next trial, based on whether the previous trial was rewarded (reward), and whether the previous trial was common or rare (transition). 'reward:transition' is the interaction between reward, and transition type, and 'intercept' refers to the intercept term. 'correct' means that whether the correct stage 1 action was taken in the previous trial. 'correct' stage 1 action in refers to the stage 1 action which led the rewarded stage 2 state. For stage 2 choices, the analysis is focused on staying on the same stage 2 action, based on staying on the same stage 1 action (stay) and earning a reward in the previous trial (reward). 'reward:stay' is the interaction between 'reward', and 'stay'. This table is different from Table S4 in two aspects: (i) the 'correct' predictor was added to the analysis following Akam et al. (2015) suggestion, (ii) unlike the analysis performed in Table S4, the trials in which subjects did not make the correct discrimination were not excluded from the analysis. Note that the aborted trial, i.e., the trials in which animals entered the magazine between stage 1 and stage 2 actions were excluded. The reason for this second difference is, since rewards are deterministic (within each reversal), if we only include trials in which animals make correct discrimination, then 'reward-transition' predictor, and 'correct' predictor will be identical. As such, in this analysis that 'correct' is a predictor, we included all the trials.

Stage 1 actions				
		probe 1	probe 2	probe 3
intercept	p-value	0.291	0.041	0.335
	β (SE)	-0.215 (0.204)	0.304 (0.149)	-0.202 (0.209)
reward	p-value	<0.001	<0.001	0.024
	β (SE)	0.418 (0.115)	0.447 (0.092)	0.390 (0.174)
transition	p-value	0.026	0.697	0.008
	β (SE)	0.365 (0.164)	0.055 (0.142)	0.614 (0.235)
correct	p-value	0.053	0.855	0.117
	β (SE)	0.566 (0.293)	0.048 (0.266)	0.399 (0.255)
reward:transition	p-value	0.625	0.777	0.026
	β (SE)	0.113 (0.233)	-0.063 (0.225)	0.527 (0.237)
Stage 2 actions				
		probe 1	probe 2	probe 3
intercept	p-value	0.156	<0.001	0.296
	β (SE)	0.153 (0.108)	0.517 (0.138)	-0.189 (0.181)
reward	p-value	0.144	<0.001	0.315
	β (SE)	0.172 (0.118)	0.568 (0.126)	0.125 (0.124)
stay	p-value	<0.001	<0.001	<0.001
	β (SE)	0.390 (0.104)	0.406 (0.110)	0.673 (0.187)
reward:stay	p-value	0.361	0.264	<0.001
	β (SE)	0.096 (0.105)	0.131 (0.118)	0.408 (0.121)
