# Supplementary material for: Learning the structure of the world: The adaptive nature of state-space and action representations in multi-stage decision-making
Source: PLoS Comput Biol. 2019 Sep 6;15(9):e1007334. doi: 10.1371/journal.pcbi.1007334 (PMC6750884; doi:10.1371/journal.pcbi.1007334)
Supplement: S6 Table — For the stage 1 choices, the analysis is focused on staying on the same stage 1 action on the next trial based on whether the previous trial was rewarded (reward) and whether the previous trial was common or rare (transition). ‘reward:transition’ is the interaction between reward, and transition type. For stage 2 choices, the analysis focuses on staying on the same stage 2 action, based on staying on the same stage 1 action (stay), and earning a reward on the previous trial (reward). ‘reward:stay’ is the interaction between ‘reward’, and ‘stay’. ‘p’ refers to p-value. For the last probe session (s94) since the pattern of choices was not stationary but changing during the session, we presented separately the analysis for the first 16 earned outcomes during the sessions (s94:1; outcomes 1:16), second 16 outcomes earned during the session (s94:2; outcomes 17:32), and third 16 outcomes earned during the session (s94:3; outcomes 33:48). (PDF) [file pcbi.1007334.s008.pdf]

Table S6. Results of the logistic regression analysis of stage 1, and stage 2 choices in supplementary experiment 1. For the stage 1 choices, the analysis is focused on staying on the same stage 1 action on the next trial based on whether the previous trial was rewarded (reward) and whether the previous trial was common or rare (transition). 'reward:transition' is the interaction between reward, and transition type. For stage 2 choices, the analysis focuses on staying on the same stage 2 action, based on staying on the same stage 1 action (stay), and earning a reward on the previous trial (reward). 'reward:stay' is the interaction between 'reward', and 'stay'. 'p' refers to p-value. For the last probe session (s94) since the pattern of choices was not stationary but changing during the session, we presented separately the analysis for the first 16 earned outcomes during the sessions (s94:1; outcomes 1:16), second 16 outcomes earned during the session (s94:2; outcomes 17:32), and third 16 outcomes earned during the session (s94:3; outcomes 33:48).

Stage 1 actions												
session		s32	S40	s49	s57	s66	s78	s87	s94	s94:1	s94:2	s94:3
intercept	p	0.773	0.312	0.017	0.299	0.177	0.162	0.885	0.428	0.46	0.131	0.194
	SE	0.151	0.11	0.16	0.124	0.136	0.11	0.228	0.127	0.248	0.263	0.29
	β	0.043	0.111	0.382	0.129	0.184	0.15	-0.032	0.1	0.183	-0.397	0.377
reward	p	0.938	0.085	0.09	0.067	0.021	0.001	0.904	<0.001	0.356	0.003	0.007
	SE	0.133	0.117	0.133	0.145	0.117	0.12	0.204	0.121	0.207	0.295	0.234
	β	-0.01	0.202	0.226	0.265	0.27	0.39	0.024	0.465	0.191	0.854	0.627
transition	p	0.764	0.628	0.098	0.016	0.825	0.383	0.993	0.498	0.407	0.644	0.335
	SE	0.14	0.105	0.191	0.119	0.124	0.13	0.24	0.14	0.187	0.296	0.185
	β	0.042	-0.051	-0.315	-0.285	-0.027	-0.11	0.002	0.094	0.155	-0.136	0.178
reward:transition	p	0.882	0.013	0.362	0.397	0.485	0.417	<0.001	<0.001	0.011	0.003	0.668
	SE	0.129	0.113	0.141	0.129	0.107	0.09	0.182	0.114	0.181	0.29	0.212
	β	0.019	-0.281	-0.129	0.109	-0.075	0.08	0.623	0.404	0.459	0.85	0.091
Stage 2 actions												
session		s32	s40	s49	s57	s66	s78	s87	s94	s94:1	s94:2	s94:3
intercept	p	<1e-7	<1e-12	<1e-11	0.963	<1e-8	<1e-14	<1e-9	<1e-16	0.969	<1e-4	0.972
	SE	0.446	0.411	0.434	190.44	0.477	0.35	0.459	0.293	195.286	0.577	514.163
	β	-2.449	-2.936	-2.996	-8.943	-2.848	-2.82	-2.969	-2.672	-7.546	-2.477	-17.949
reward	p	0.933	0.938	0.148	0.984	0.383	0.137	0.169	0.019	0.98	0.924	0.995
	SE	0.327	0.379	0.459	190.461	0.433	0.32	0.459	0.268	195.285	0.512	740.305
	β	0.027	0.029	0.662	3.825	0.377	0.48	0.631	0.626	5.015	0.049	4.749
stay	p	0.057	0.852	0.074	0.973	0.401	0.386	0.891	0.798	0.98	0.387	0.994
	SE	0.406	0.522	0.487	190.44	0.405	0.33	0.463	0.278	195.286	0.507	665.334
	β	0.771	-0.097	0.868	-6.429	0.339	0.29	0.063	0.071	-4.891	0.438	5.35
reward:stay	p	0.105	0.515	0.957	0.982	0.089	0.2	0.443	0.853	0.984	0.809	0.994
	SE	0.32	0.356	0.454	190.46	0.398	0.37	0.49	0.283	195.286	0.503	615.096
	β	0.519	0.232	-0.024	4.252	0.677	0.47	0.376	-0.052	3.974	-0.121	4.881
