# Supplementary material for: Learning the structure of the world: The adaptive nature of state-space and action representations in multi-stage decision-making
Source: PLoS Comput Biol. 2019 Sep 6;15(9):e1007334. doi: 10.1371/journal.pcbi.1007334 (PMC6750884; doi:10.1371/journal.pcbi.1007334)
Supplement: S7 Table — (PDF) [file pcbi.1007334.s009.pdf]

Table S7. Total number of trials completed by the subjects in the supplementary experiment 1.

session	mean (SD)
s32	90.000 (9.381)
s40	91.500 (8.036)
s49	88.625 (13.585)
s57	82.250 (13.392)
s66	86.000 (10.142)
s78	94.500 (4.957)
s87	87.375 (8.749)
s94	92.625 (9.486)
