# Supplementary material for: Learning the structure of the world: The adaptive nature of state-space and action representations in multi-stage decision-making
Source: PLoS Comput Biol. 2019 Sep 6;15(9):e1007334. doi: 10.1371/journal.pcbi.1007334 (PMC6750884; doi:10.1371/journal.pcbi.1007334)
Supplement: S8 Table — For the stage 1 choices, the analysis is focused on staying on the same stage 1 action on the next trial, based on whether the previous trial was rewarded (reward), and whether the previous trial was common or rare (transition). ‘reward:transition’ is the interaction between reward, and transition type. For stage 2 choices, the analysis is focused on staying on the same stage 2 action, based on staying on the same stage 1 action (stay) and earning a reward in the previous trial (reward). ‘reward:stay’ is the interaction between ‘reward’, and ‘stay’. (PDF) [file pcbi.1007334.s010.pdf]

Table S8. Results of the logistic regression analysis of stage 1 and stage 2 choices in supplementary experiments 2 and 3. For the stage 1 choices, the analysis is focused on staying on the same stage 1 action on the next trial, based on whether the previous trial was rewarded (reward), and whether the previous trial was common or rare (transition). 'reward:transition' is the interaction between reward, and transition type. For stage 2 choices, the analysis is focused on staying on the same stage 2 action, based on staying on the same stage 1 action (stay) and earning a reward in the previous trial (reward). 'reward:stay' is the interaction between 'reward', and 'stay'.

Stage 1 actions			
		Supplementary experiment 2	Supplementary experiment 3
session		s76	s74
intercept	*p*-value	<1e-8	0.74
	β (SE)	0.78 (0.134)	-0.042 (0.128)
reward	*p*-value	<0.001	<1e-7
	β (SE)	0.451 (0.132)	0.509 (0.094)
transition	*p*-value	0.467	0.726
	β (SE)	-0.11 (0.152)	-0.055 (0.159)
reward:transition	*p*-value	0.19	0.485
	β (SE)	0.183 (0.14)	0.066 (0.095)
**Stage 2 actions**			
		Supplementary experiment 2	Supplementary experiment 3
session		s76	s74
intercept	*p*-value	<1e-13	<1e-15
	β (SE)	-2.611 (0.35)	-0.863 (0.093)
reward	*p*-value	0.145	0.002
	β (SE)	0.56 (0.384)	0.276 (0.091)
stay	*p*-value	0.887	0.014
	β (SE)	-0.047 (0.336)	0.235 (0.096)
reward:stay	*p*-value	0.6	0.44
	β (SE)	0.182 (0.348)	0.065 (0.085)
