# Supplementary material for: Learning the structure of the world: The adaptive nature of state-space and action representations in multi-stage decision-making
Source: PLoS Comput Biol. 2019 Sep 6;15(9):e1007334. doi: 10.1371/journal.pcbi.1007334 (PMC6750884; doi:10.1371/journal.pcbi.1007334)
Supplement: S9 Table — (PDF) [file pcbi.1007334.s011.pdf]

Table S9. Total number of trials completed by the subjects in the supplementary experiments 2, 3

session	mean (SD)
Supplementary experiment 2	88 (10.736)
Supplementary experiment 3	123 (13.093)
