# Supplementary material for: Learning the structure of the world: The adaptive nature of state-space and action representations in multi-stage decision-making
Source: PLoS Comput Biol. 2019 Sep 6;15(9):e1007334. doi: 10.1371/journal.pcbi.1007334 (PMC6750884; doi:10.1371/journal.pcbi.1007334)

Figure S1. The graph shows the intercept term for the analysis shown in Figure 3a, which is the log odds ratio of staying on the same stage 1 action. The *p*-value of this analysis (for the intercept term) was used for colour-coding each bar. The graphs can be interpreted as the tendency of staying on the same stage 1 action, independent of whether reward was earn in the previous trial. The sessions marked with 'strict sequence' are similar to the sessions described in Figure 3a.

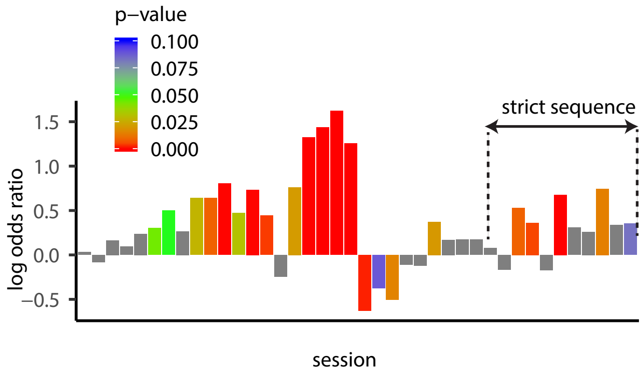

Supplement: S1 Fig — The p-value of this analysis (for the intercept term) was used for colour-coding each bar. The graphs can be interpreted as the tendency of staying on the same stage 1 action, independent of whether reward was earn in the previous trial. The sessions marked with ‘strict sequence’ are similar to the sessions described in Fig 3a. (PDF) [file pcbi.1007334.s012.pdf]
