# Supplementary material for: Learning the structure of the world: The adaptive nature of state-space and action representations in multi-stage decision-making
Source: PLoS Comput Biol. 2019 Sep 6;15(9):e1007334. doi: 10.1371/journal.pcbi.1007334 (PMC6750884; doi:10.1371/journal.pcbi.1007334)

Figure S2. The graph shows the average session duration (across subjects). The animals could earn maximum of 60 outcomes and the session ended as soon as animals earned 60 outcomes. The session length was limited to an hour. The results are for the experiment reported in the main paper. Error-bars represent 1SEM.

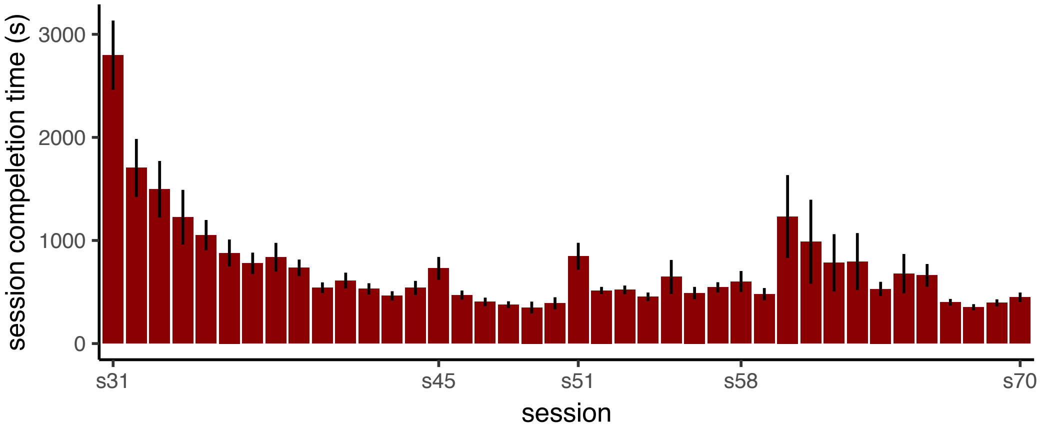

Supplement: S2 Fig — The animals could earn maximum of 60 outcomes and the session ended as soon as animals earned 60 outcomes. The session length was limited to an hour. The results are for the experiment reported in the main paper. Error-bars represent 1SEM. (PDF) [file pcbi.1007334.s013.pdf]
