# Supplementary material for: Learning the structure of the world: The adaptive nature of state-space and action representations in multi-stage decision-making
Source: PLoS Comput Biol. 2019 Sep 6;15(9):e1007334. doi: 10.1371/journal.pcbi.1007334 (PMC6750884; doi:10.1371/journal.pcbi.1007334)

Figure S3. Log odds ratio of the probability of staying on the same stage 1 action after getting rewarded on the previous trial (for the main experiment). Each panel shows the data for each subject. Log odds ratio=0 implies an equal preference for both actions. The odds ratios are calculated using logistic regression and p-values are colour coded.

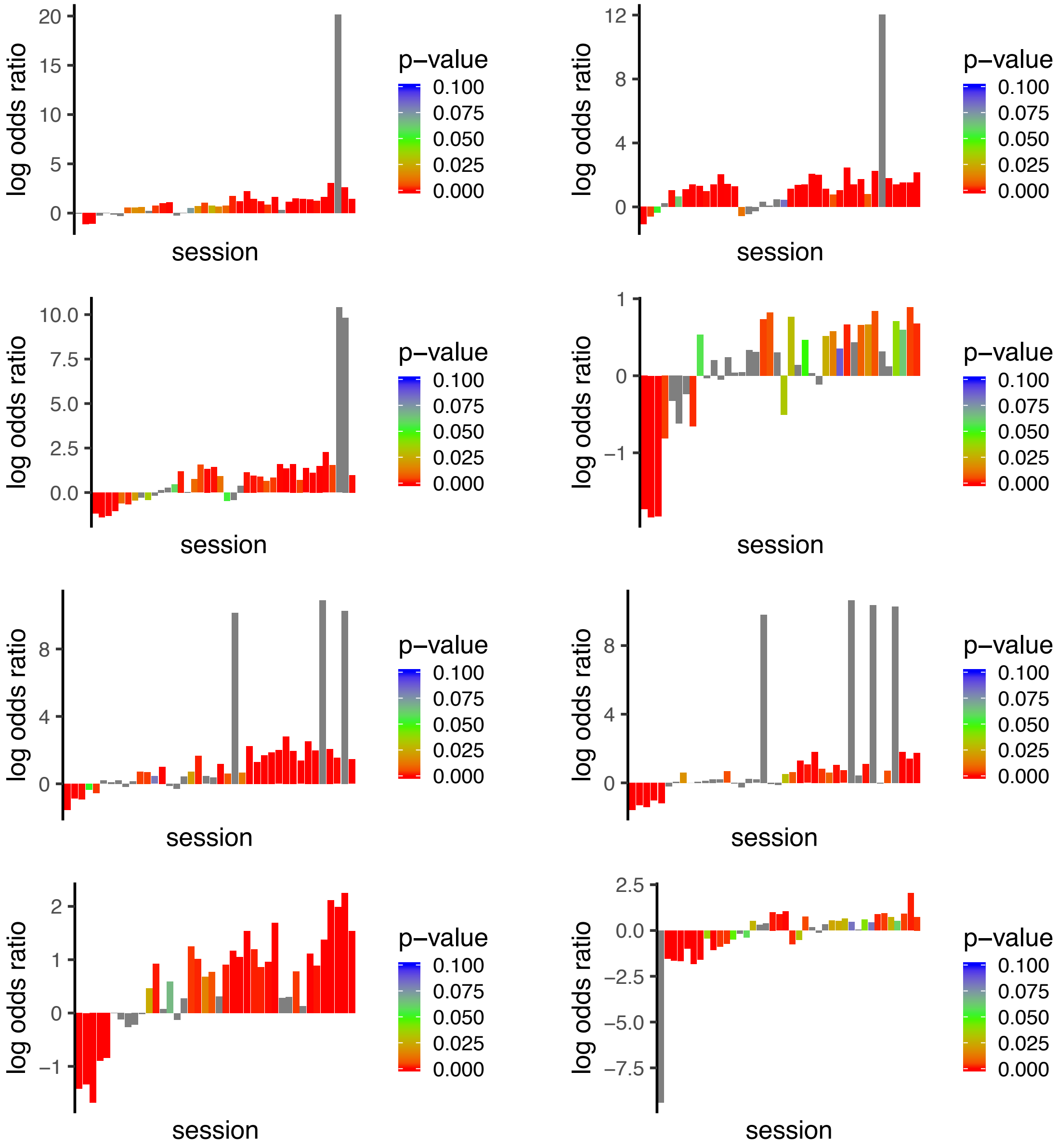

Supplement: S3 Fig — Each panel shows the data for each subject. Log odds ratio = 0 implies an equal preference for both actions. The odds ratios are calculated using logistic regression and p-values are colour coded. (PDF) [file pcbi.1007334.s014.pdf]
