# Supplementary material for: Learning the structure of the world: The adaptive nature of state-space and action representations in multi-stage decision-making
Source: PLoS Comput Biol. 2019 Sep 6;15(9):e1007334. doi: 10.1371/journal.pcbi.1007334 (PMC6750884; doi:10.1371/journal.pcbi.1007334)

Figure S4. Results of discrimination training (for the experiment reported in the main paper) showing the percentage of correct responses averaged over subjects. Each point refers to a training session and error-bars are ±1 SEM.

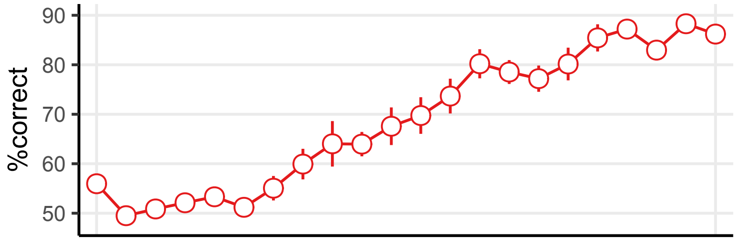

Supplement: S4 Fig — Each point refers to a training session and error-bars are ±1 SEM. (PDF) [file pcbi.1007334.s015.pdf]
