# Supplementary material for: Learning the structure of the world: The adaptive nature of state-space and action representations in multi-stage decision-making
Source: PLoS Comput Biol. 2019 Sep 6;15(9):e1007334. doi: 10.1371/journal.pcbi.1007334 (PMC6750884; doi:10.1371/journal.pcbi.1007334)

Figure S5. Supplementary experiment 1. In this experiment several probe sessions were inserted in the middle of the training sessions in order illustrate the development of actions over the training period. (a) Log odds ratio of the probability of staying on the same stage 1 action after getting rewarded on the previous trial. Log odds ratio=0 implies an equal preference for both actions. Sessions marked with '*' are the probe sessions which included both rare and common transitions. (b-f) The probability of staying on the same stage 1 action in the probe sessions, averaged over subjects, as a function of whether the previous trial was rewarded (reward/no reward), and whether the transition in the previous trial was common or rare. The graphs illustrate a gradual shift from a simple state-space representation (panel b) to reward-guided actions (panels c,d), to goal-directed choices (panel e), and finally to a mixture of goal-directed and automatic actions (panel f). (g) Stage 2 actions in session s78. The graph shows the probability of staying on the same stage 2 action, averaged over subjects, as a function of whether the previous trial was rewarded (reward/no reward), and whether subjects stayed on the same stage 1 action (stay/switch). Similar to the analysis of the experiment reported in the main paper, only trials in which the stage 2 state is different from the previous trial are included in panel (g) in order to detect the performance of action sequences. Similarly, only trials in which subjects made a correct discrimination on the previous trial ('R' in S2, and 'L' in S1) were included in panels (a-g; see text). In all the probe sessions, the probability of rare transitions was 80%, except for the last session (s94) in which the probability of common and rare transitions was equal (i.e., 50%), in order to establish the effect of the transition probabilities on actions. (h) Results of initial discrimination training showing the percentage of correct responses averaged over subjects. Error-bars ±1 SEM.

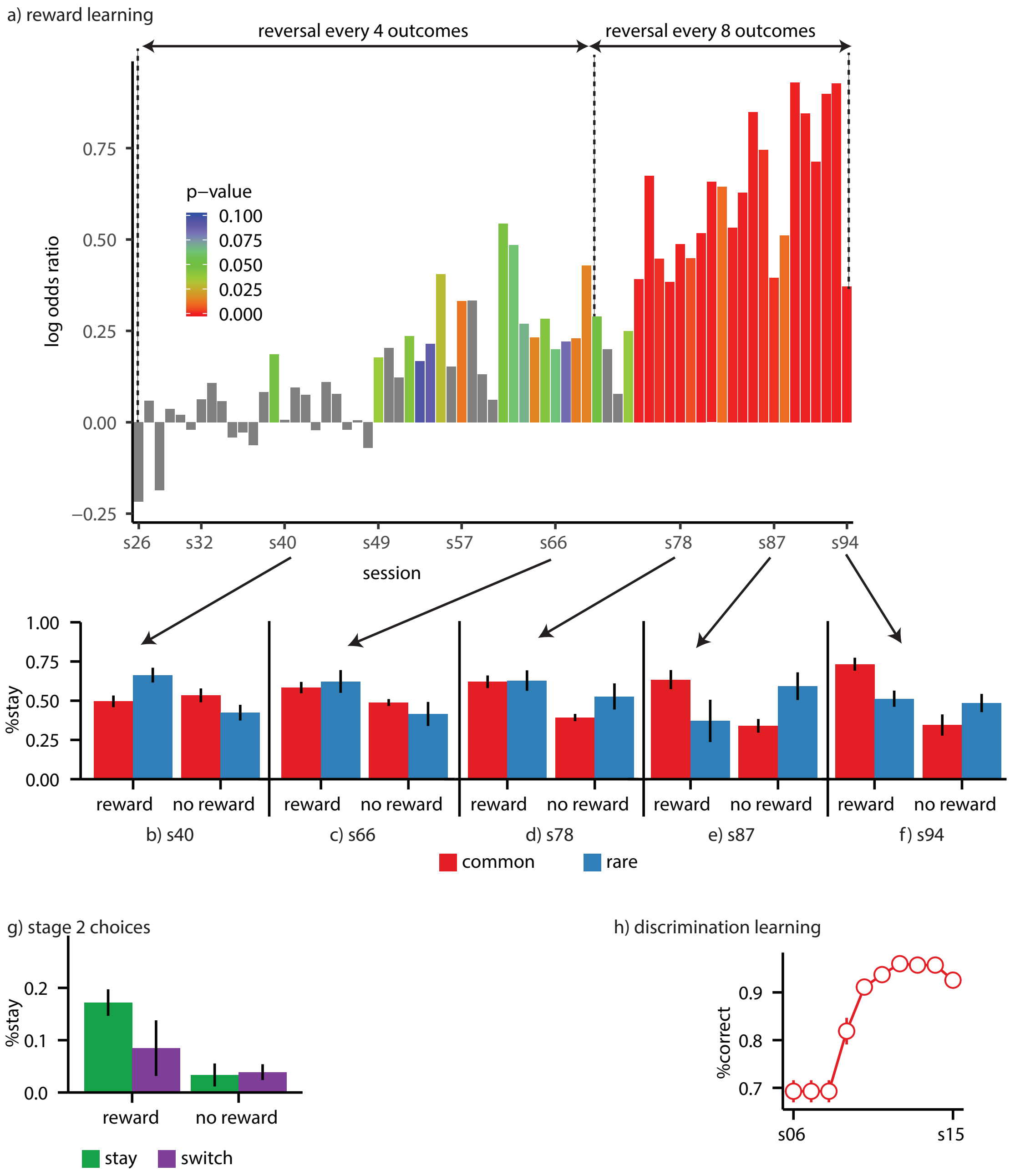

Supplement: S5 Fig — In this experiment several probe sessions were inserted in the middle of the training sessions in order illustrate the development of actions over the training period. (a) Log odds ratio of the probability of staying on the same stage 1 action after getting rewarded on the previous trial. Log odds ratio = 0 implies an equal preference for both actions. Sessions marked with ‘*’ are the probe sessions which included both rare and common transitions. (b-f) The probability of staying on the same stage 1 action in the probe sessions, averaged over subjects, as a function of whether the previous trial was rewarded (reward/no reward), and whether the transition in the previous trial was common or rare. The graphs illustrate a gradual shift from a simple state-space representation (panel b) to reward-guided actions (panels c,d), to goal-directed choices (panel e), and finally to a mixture of goal-directed and automatic actions (panel f). (g) Stage 2 actions in session s78. The graph shows the probability of staying on the same stage 2 action, averaged over subjects, as a function of whether the previous trial was rewarded (reward/no reward), and whether subjects stayed on the same stage 1 action (stay/switch). Similar to the analysis of the experiment reported in the main paper, only trials in which the stage 2 state is different from the previous trial are included in panel (g) in order to detect the performance of action sequences. Similarly, only trials in which subjects made a correct discrimination on the previous trial (‘R’ in S2, and ‘L’ in S1) were included in panels (a-g; see text). In all the probe sessions, the probability of rare transitions was 80%, except for the last session (s94) in which the probability of common and rare transitions was equal (i.e., 50%), in order to establish the effect of the transition probabilities on actions. (h) Results of initial discrimination training showing the percentage of correct responses averaged over subjects. Error-bars ± [file pcbi.1007334.s016.pdf]
