# Supplementary material for: Learning the structure of the world: The adaptive nature of state-space and action representations in multi-stage decision-making
Source: PLoS Comput Biol. 2019 Sep 6;15(9):e1007334. doi: 10.1371/journal.pcbi.1007334 (PMC6750884; doi:10.1371/journal.pcbi.1007334)

Figure S6. Supplementary experiment 2. (a) The log odds ratio of staying on the same stage 1 action after earning a reward on the previous trial over the odds after earning no reward. Sessions denoted by 'all rare' included only rare transitions (similar to sessions marked with '#' in Figure 3a). (b) The probability of staying on the same stage 1 action in the probe session (session s76) as a function of whether the previous trial was rewarded (reward/no reward) and whether the transition in the previous trial was common or rare. (c) The probability of staying on the same stage 2 action in the probe session (session s76), as a function of whether the previous trial was rewarded (reward/no reward) and whether subjects stayed on the same stage 1 action (stay/switch). Similar to the analysis in the main paper, only trials in which the stage 2 state was different from the previous trial are included in panels (c) in order to detect the performance of action sequences. Similarly, only trials in which subjects made a correct discrimination on the previous trial ('R' in S2, and 'L' in S1) were included in panels (a-c). In all the probe sessions the probability of rare transitions was 50%. (d) Results of discrimination training showing the percentage of correct responses. Error-bars ±1 SEM.

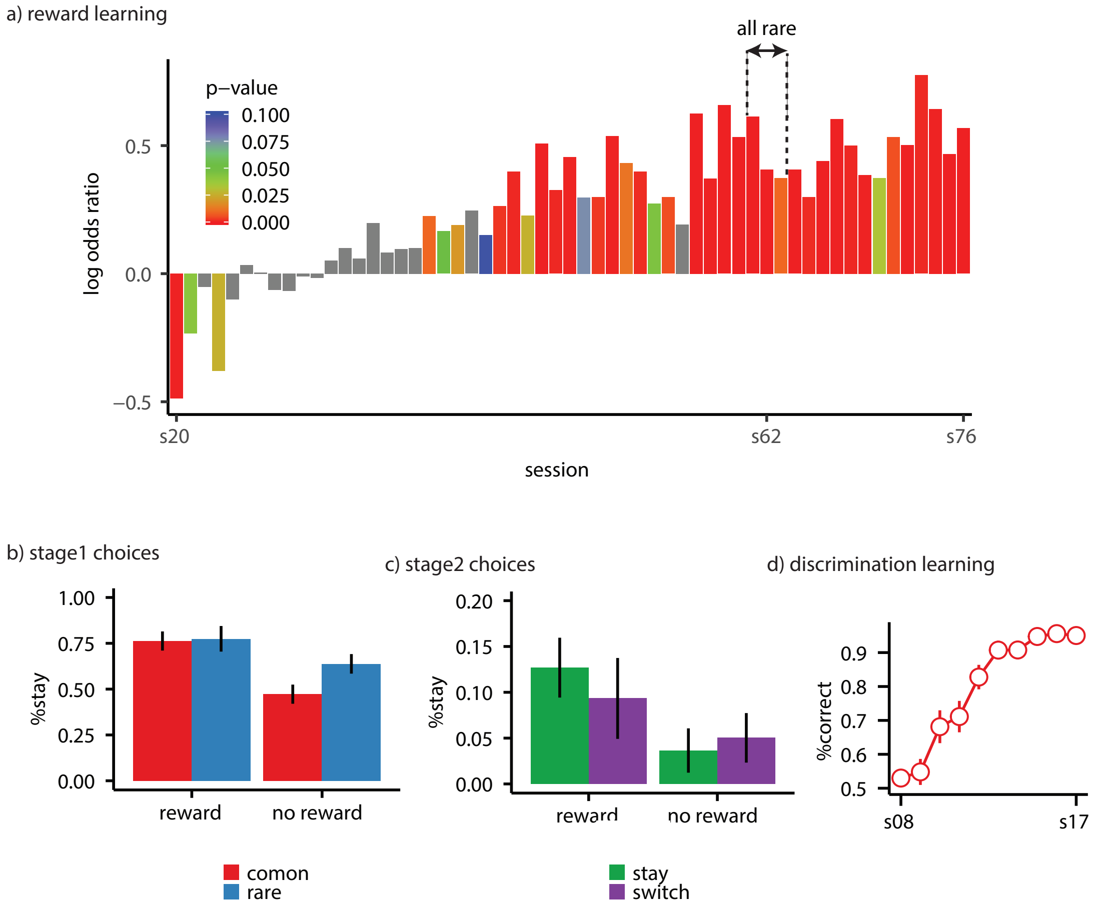

Supplement: S6 Fig — (a) The log odds ratio of staying on the same stage 1 action after earning a reward on the previous trial over the odds after earning no reward. Sessions denoted by ‘all rare’ included only rare transitions (similar to sessions marked with ‘#’ in Fig 3a). (b) The probability of staying on the same stage 1 action in the probe session (session s76) as a function of whether the previous trial was rewarded (reward/no reward) and whether the transition in the previous trial was common or rare. (c) The probability of staying on the same stage 2 action in the probe session (session s76), as a function of whether the previous trial was rewarded (reward/no reward) and whether subjects stayed on the same stage 1 action (stay/switch). Similar to the analysis in the main paper, only trials in which the stage 2 state was different from the previous trial are included in panels (c) in order to detect the performance of action sequences. Similarly, only trials in which subjects made a correct discrimination on the previous trial (‘R’ in S2, and ‘L’ in S1) were included in panels (a-c). In all the probe sessions the probability of rare transitions was 50%. (d) Results of discrimination training showing the percentage of correct responses. Error-bars ±1 SEM. (PDF) [file pcbi.1007334.s017.pdf]
