# Supplementary material for: Learning the structure of the world: The adaptive nature of state-space and action representations in multi-stage decision-making
Source: PLoS Comput Biol. 2019 Sep 6;15(9):e1007334. doi: 10.1371/journal.pcbi.1007334 (PMC6750884; doi:10.1371/journal.pcbi.1007334)

Figure S7. Supplementary experiment 3. (a) The log odds ratio of staying on the same stage 1 action after earning a reward on the previous trial over the odds after earning no reward. Sessions denoted by 'all rare' included only rare transitions (similar to sessions marked with '#' in Figure 3a). 'strict sequences' indicates that trials with magazine responses after stage 1 actions were aborted. (b) The probability of staying on the same stage 1 action in the probe session (session s74) as a function of whether the previous trial was rewarded (reward/no reward), and whether the transition in the previous trial was common or rare. (c) The probability of staying on the same stage 2 action in the probe session (session s74), as a function of whether the previous trial was rewarded (reward/no reward), and whether subjects stayed on the same stage 1 action (stay/switch). Similar to the analysis presented in the main paper, only trials in which the stage 2 states were different from the previous trial are included in panels (c) in order to detect the performance of action sequences. Similar to the analysis in the main paper, only trials in which subjects made a correct discrimination on the previous trial ('R' in S2, and 'L' in S1) were included in panels (a-c). In the probe sessions, the probability of rare transitions was 50%. (d) Results of discrimination training showing the percentage of correct responses. Error-bars ±1 SEM.

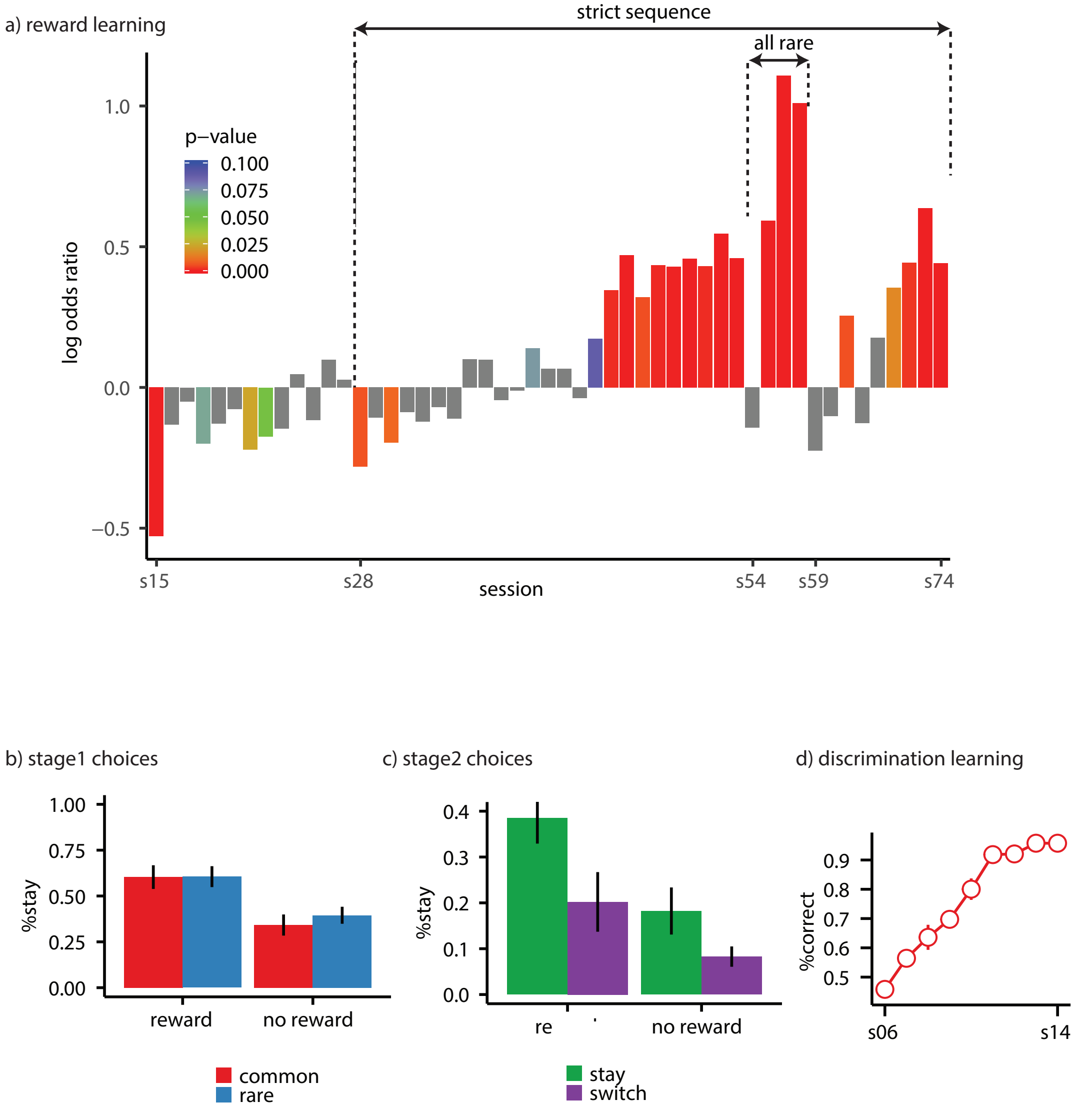

Supplement: S7 Fig — (a) The log odds ratio of staying on the same stage 1 action after earning a reward on the previous trial over the odds after earning no reward. Sessions denoted by ‘all rare’ included only rare transitions (similar to sessions marked with ‘#’ in Fig 3a). ‘strict sequences’ indicates that trials with magazine responses after stage 1 actions were aborted. (b) The probability of staying on the same stage 1 action in the probe session (session s74) as a function of whether the previous trial was rewarded (reward/no reward), and whether the transition in the previous trial was common or rare. (c) The probability of staying on the same stage 2 action in the probe session (session s74), as a function of whether the previous trial was rewarded (reward/no reward), and whether subjects stayed on the same stage 1 action (stay/switch). Similar to the analysis presented in the main paper, only trials in which the stage 2 states were different from the previous trial are included in panels (c) in order to detect the performance of action sequences. Similar to the analysis in the main paper, only trials in which subjects made a correct discrimination on the previous trial (‘R’ in S2, and ‘L’ in S1) were included in panels (a-c). In the probe sessions, the probability of rare transitions was 50%. (d) Results of discrimination training showing the percentage of correct responses. Error-bars ±1 SEM. (PDF) [file pcbi.1007334.s018.pdf]
