# Supplementary figures and images for: Learning the structure of the world: The adaptive nature of state-space and action representations in multi-stage decision-making
Source: PLoS Comput Biol. 2019 Sep 6;15(9):e1007334. doi: 10.1371/journal.pcbi.1007334 (PMC6750884; doi:10.1371/journal.pcbi.1007334)

stage 2 actions (simulated)

stage 1 actions (simulated)

HMB

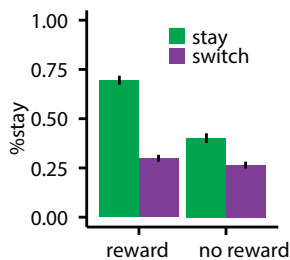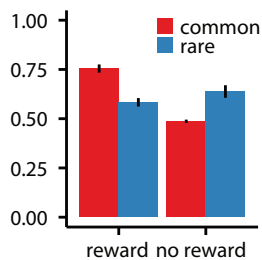

MF

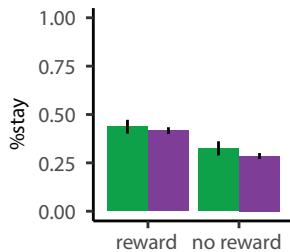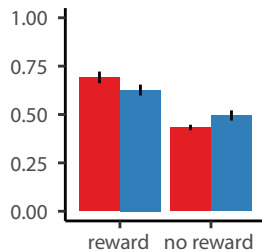

MB

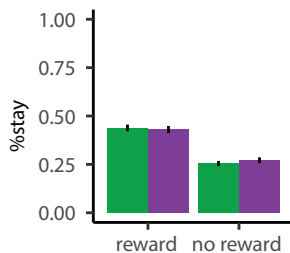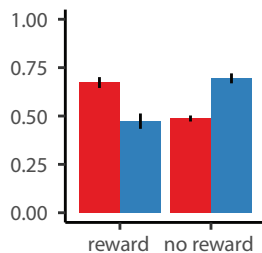

Supplement: S8 Fig — Note that similar to the other figures, in the left column only the trials in which state 2 state is different from the previous trial are included. (PDF) [file pcbi.1007334.s019.pdf]
